# A comprehensive survey of AI agents in healthcare

**DOI:** 10.1016/j.jbi.2026.105045

**Published:** 2026-04-18

**Authors:** Gelei Xu, Xueyang Li, Yixiong Chen, Yuying Duan, Shuqing Wu, Haoxinran Yu, Ching-Hao Chiu, Juntong Ni, Ningzhi Tang, Toby Jia-Jun Li, Alan Yuille, Wei Jin, Yiyu Shi

**Affiliations:** a University of Notre Dame, Notre Dame, IN 46556, USA; b Johns Hopkins University, Baltimore, MD 21218, USA; c Emory University, Atlanta, GA 30322, USA

**Keywords:** Agentic AI, Multi-agent systems, Clinical decision support, Medical reasoning

## Abstract

**Objective:**

This survey aims to systematically map the rapidly evolving landscape of AI agents in healthcare. It addresses the critical need to adapt general-purpose agentic frameworks characterized by autonomy, planning, and tool use to the high-stakes, safety-critical constraints of medical decision-making and patient care.

**Methods:**

We conducted a comprehensive review of over 200 recent studies, synthesizing literature from major academic databases. We developed a holistic taxonomy that traces the full lifecycle of healthcare agents, analyzing perception modalities, core technical architectures, and evaluation protocols specific to autonomous systems.

**Results:**

The review presents a quantitative landscape analysis showing exponential growth in the field. We structure the domain into three pillars: (1) *Perception* of multi-modal clinical data (e.g., EHR, imaging, genomics); (2) *Agent Capabilities*, including tool use, reasoning, memory, and multi-agent collaboration; and (3) an *Application Ecosystem* organized by stakeholder roles (clinicians, patients, researchers, and administrators). Additionally, we categorize evaluation frameworks, and discuss the deployment readiness of current systems across technical, evidentiary, and governance dimensions. Finally, we identify challenges for advancing healthcare agents from controlled evaluation toward real-world clinical integration. A continuously updated repository of related papers is available at https://github.com/AgenticHealthAI/Awesome-AI-Agents-for-Healthcare.

**Conclusion:**

AI agents offer significant potential to enhance healthcare through autonomous reasoning and workflow integration. However, current research remains largely concentrated in benchmark and controlled evaluation settings, and the translation into clinical practice will require advances in reliability, privacy protection, governance, and operational integration.

## 1. Introduction

The emergence of large language models (LLMs) has advanced AI systems toward the capabilities of artificial general intelligence [1]. A key development in this progression is the rise of AI agents [2,3], which extends predictive modeling structured autonomy, planning, memory, and tool use. Unlike single-pass generation systems, agents can pursue goals through multi-step reasoning, incorporate prior interaction history, and invoke external tools to support task execution. These capabilities allow AI systems to function in dynamic and complex environments beyond narrowly predefined tasks.

Healthcare presents a strong demand for AI agents. It is a knowledge-intensive domain in which clinicians manage heterogeneous sources such as electronic health records (EHRs), imaging, genomics, and medical literature [4–6]. Agents can integrate fragmented inputs into longitudinal patient profiles through tools and memory. Many clinical tasks also require long-horizon, adaptive decision-making. For instance, cancer management, chronic disease care, and acute interventions involve iterative planning and adjustment to evolving patient states [7]. Fragmented workflows and rising expectations for personalized communication further underscore the role of agents as workflow coordinators [8]. By managing analysis, documentation, and communication while maintaining continuity through memory and persona conditioning, agentic systems can act as integrated partners in care delivery.

Despite these prospects, the adoption of AI agents in clinical practice remains more limited than in many other domains. Healthcare is a highly stakes and tightly regulated environment where errors directly affect patient safety [9,10]. The autonomy that characterizes agentic systems therefore raises concerns regarding safety, oversight, and legal accountability. Clinical systems that access sensitive data such as EHRs, imaging, and patient dialogues must comply with strict regulatory requirements (e.g., HIPAA, GDPR) [11–13]. Integration of multimodal data must preserve privacy while supporting clinically meaningful outputs. Clinical decision support also carries ethical obligations that extend beyond predictive accuracy. Mechanisms for transparency and human oversight are required to enable clinician review and preserve final authority [14,15]. These requirements show that healthcare agents cannot simply replicate general-purpose designs [16]. The central design problem lies in ensuring safety, accountability, and effective performance under uncertainty.

Research on healthcare agents has expanded rapidly. This growth parallels advances in tool-use language models [17], multimodal foundation models [18,19], and interoperable clinical APIs [20]. These developments enable agents to access clinical data, coordinate tasks, and operate within controlled environments with increasing reliability. Despite this progress, systematic analyses that connect these technical advances to the specific constraints of healthcare remain limited. Existing surveys [21] summarize architectures and applications but cover a relatively narrow body of work and provide limited examination of how modality characteristics and system design interact with domain-specific requirements. This survey provides the first comprehensive synthesis, analyzing over 200 recent studies and integrating perspectives across perception, agent capabilities, and application domains to offer a unified view of how healthcare agents are designed, evaluated, and translated toward deployment.

The remainder of this paper is organized as follows. Section 2 presents a macro-level analysis of current trends in healthcare agent development, and Section 3 outlines the survey methodology. Section 4 discusses perception and environment. Section 5 examines the core agent architectures and technical capabilities. Section 6 reviews applications across the healthcare ecosystem organized by key stakeholders. Section 7 focuses on the evaluation of current agentic systems. Section 8 examines deployment readiness of current agentic frameworks. Section 9 discusses open challenges and future directions. Section 10 concludes.

In summary, this survey structures recent work on AI agents in healthcare into a comprehensive framework (Table 1). For computer scientists, it outlines how safety-critical constraints in healthcare influence autonomy, evaluation, and system design. For clinicians, it clarifies emerging applications and current limitations to inform adoption and oversight. We maintain a continuously updated repository of related papers at link to support ongoing research.

### Connections to Existing Surveys.

Several recent surveys analyze aspects of AI agents in healthcare. Some review selected systems and focus on planning strategies or ethical considerations [21,22]. Others target specific domains such as radiology or outline implementation roadmaps that emphasize privacy and interoperability [23,24]. Broader surveys of language models and autonomous agents discuss architectures, datasets, and evaluation practices across fields [2,25], with limited coverage of safety-critical and regulatory factors unique to healthcare. Building on these contributions, this survey synthesizes more than 200 recent studies and introduces a taxonomy connecting perception modalities, agent capabilities, stakeholder applications, and evaluation practice. By situating technical advances within clinical and regulatory contexts, it provides a domain-specific synthesis that complements prior reviews.

## 2. A landscape of AI agents in healthcare

### **Operational Definition of Healthcare Agents**.

This review focuses on systems designed to support tasks situated within healthcare delivery, biomedical research, patient management, or public health contexts. We considered an application healthcare-relevant if its stated purpose involved diagnosis, triage, treatment planning, clinical documentation, patient interaction, care coordination, medical education, or related workflows. General-purpose demonstrations without a concrete healthcare objective were excluded. We define a healthcare agent as a system that extends beyond single-pass model inference by incorporating explicit mechanisms for iterative control or action selection in pursuit of such tasks. Importantly, the designation of an agent was based on system-level design rather than intrinsic model capabilities. A study was considered agentic if it reported at least one of the following properties: (1) multi-step or looped execution where intermediate outputs influenced subsequent operations; (2) dynamic invocation of external tools, databases, or APIs; (3) maintenance of task-relevant state or memory across steps; or (4) coordination among multiple roles or modules with delegated responsibilities. We excluded systems limited to static prediction, single-turn question answering, or passive text generation without adaptive control logic. For example, prompting a general-purpose language model to answer a medical question was not sufficient for inclusion unless the system additionally implemented explicit orchestration, planning, or tool-mediated interaction.

### 2.1. A conceptual framework for AI agents in healthcare

**Fig. 1.**
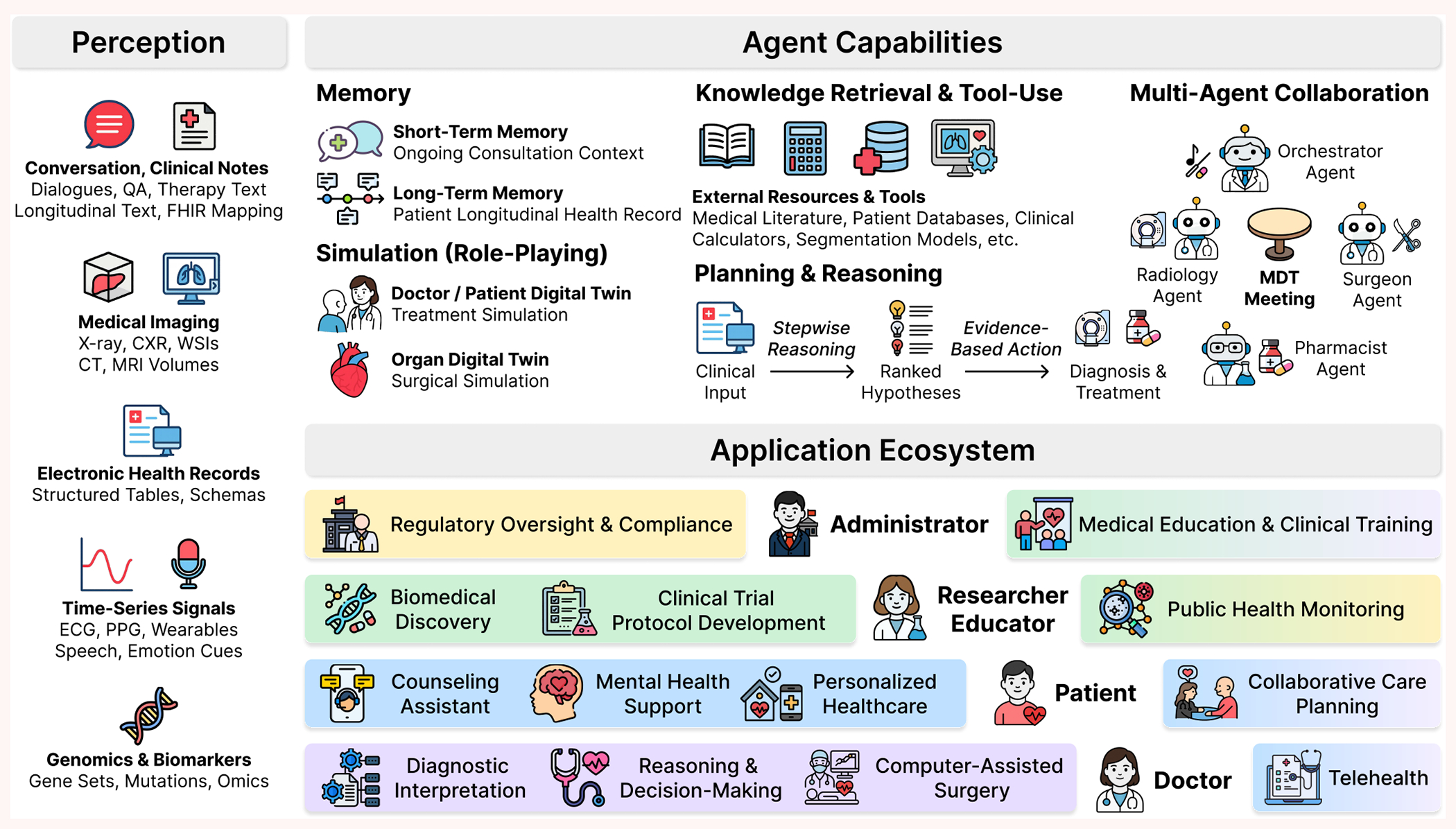
A conceptual framework for AI agents in healthcare. The framework illustrates the flow from data **Perception** and core **Agent Capabilities** to a hierarchical **Healthcare Agent Application Ecosystem**, organized around four central stakeholders (doctor, patient, researcher/educator, and administrator), each linked to both role-specific and collaborative applications.

To organize the emerging ecosystem of AI agents in healthcare, we introduce a conceptual framework shown in Fig. 1. The framework is built upon three core pillars: **Perception** represents the clinical inputs available to agents, including structured EHR data, unstructured text and medical images, genomic information, and time-series signals. These modalities define the information basis for agent behavior. **Agent Capabilities** capture the foundational mechanisms that enable agentic behavior, including memory, tool integration, planning and reasoning, simulation, and multi-agent coordination. **Application Ecosystem **organizes healthcare use cases by stakeholder roles, including clinicians, patients, researchers, educators, and administrators, as well as cross-stakeholder workflows that require coordinated interaction. This perspective reflects the operational structure of healthcare systems.

### 2.2. Quantitative analysis of the research landscape

**Fig. 2.**
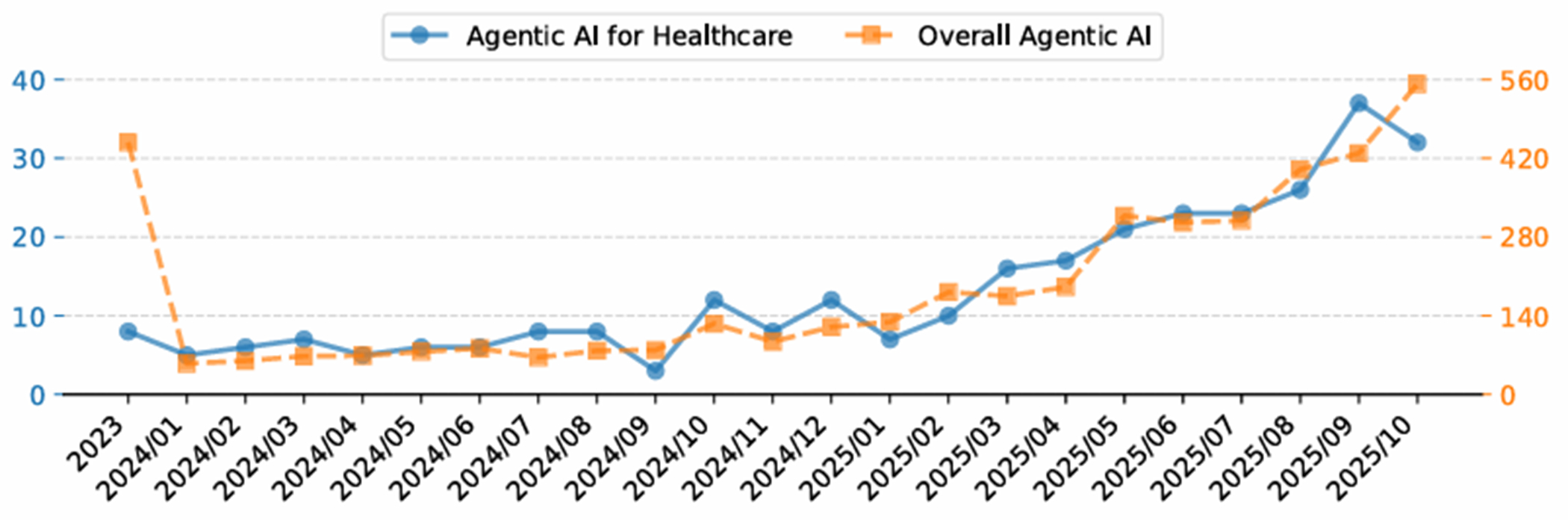

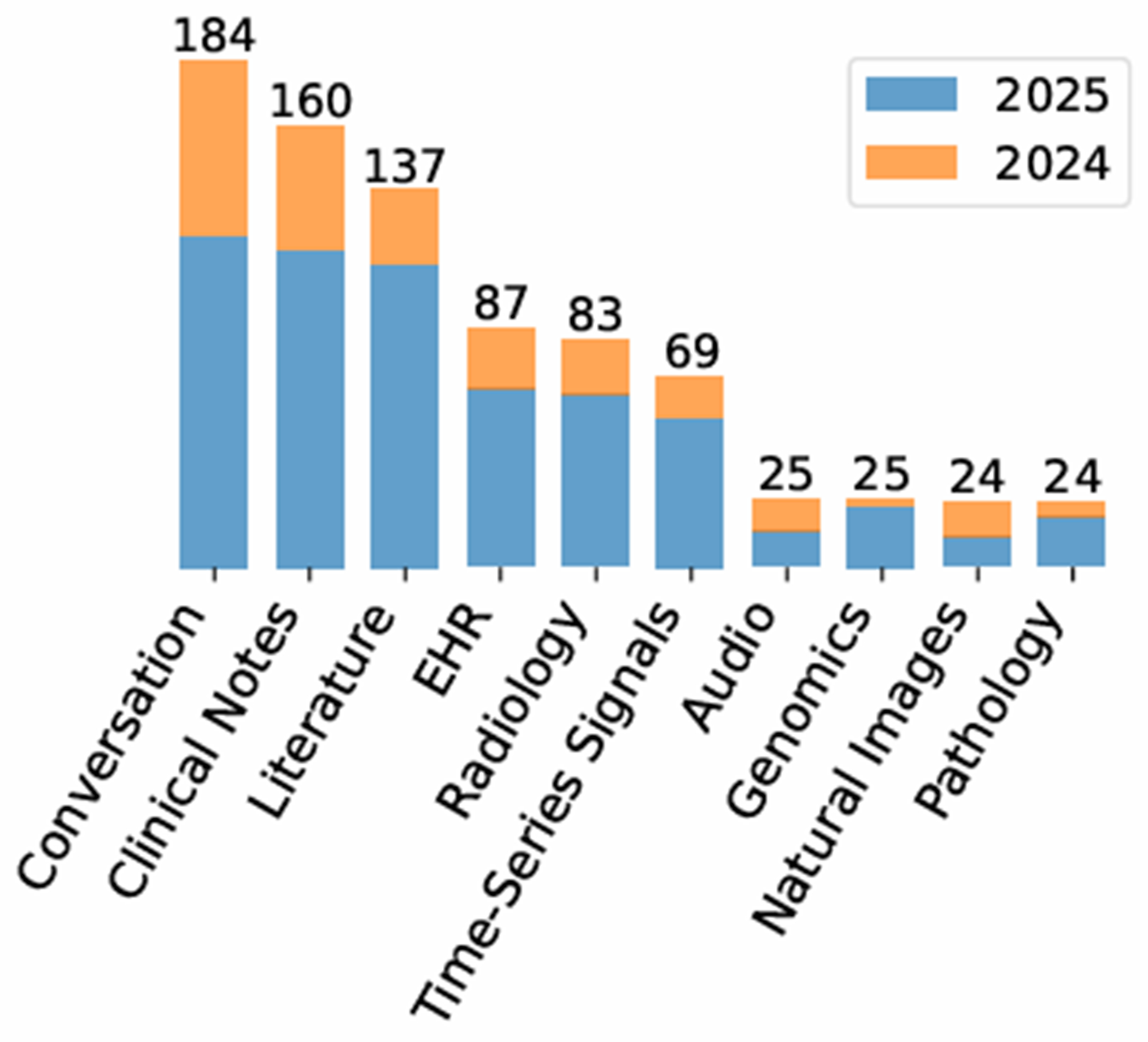

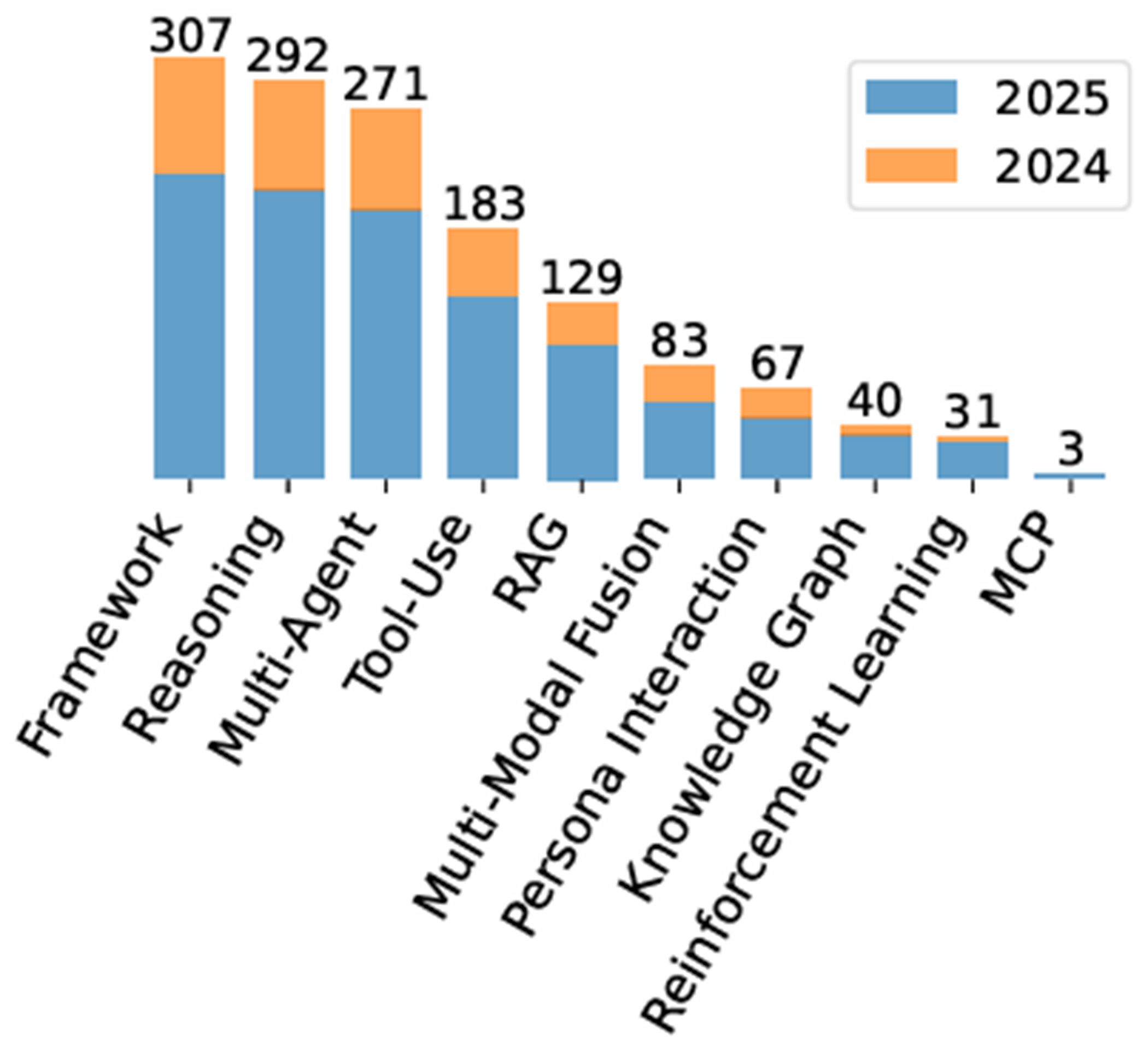

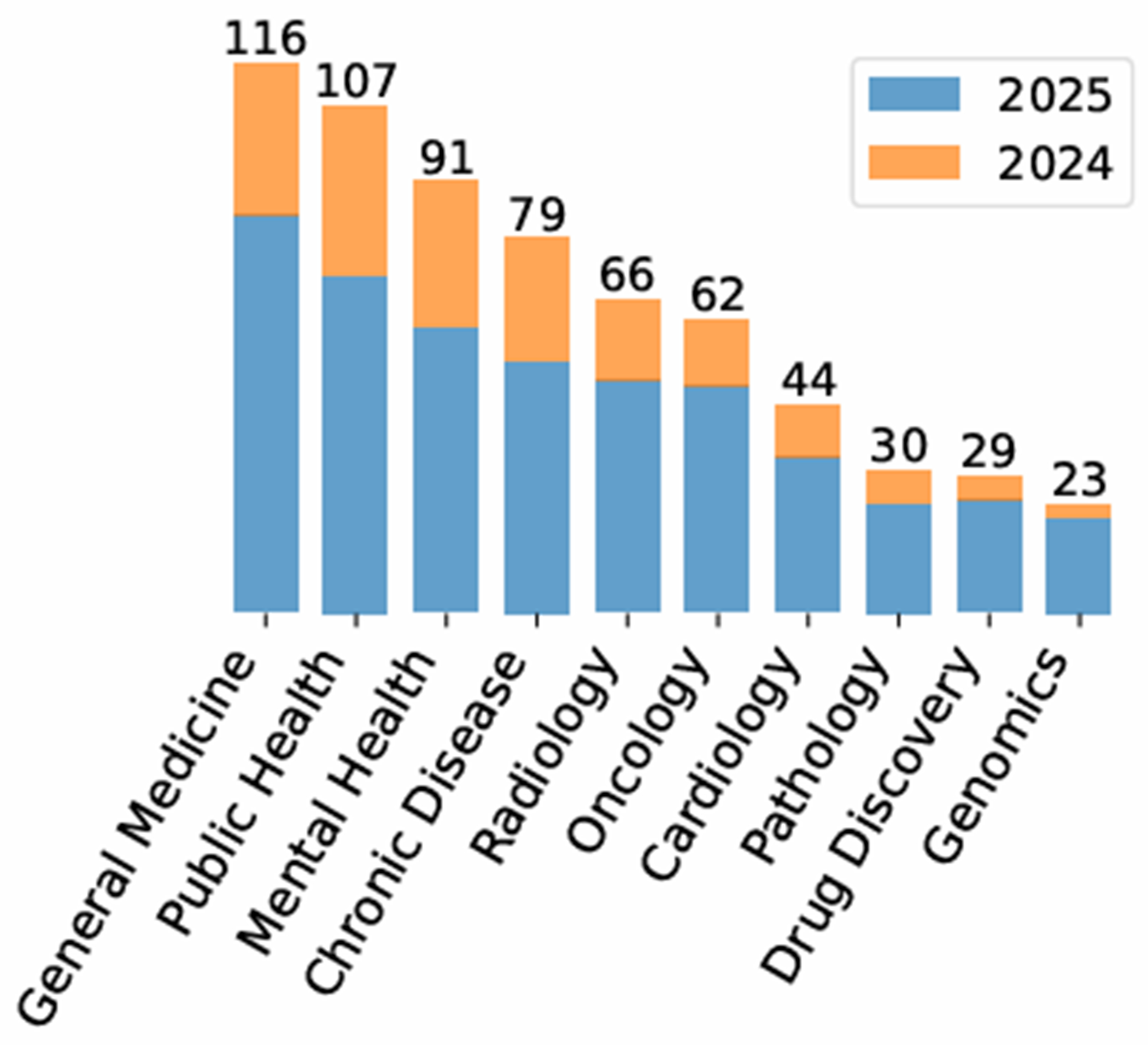
Quantitative analysis of the research landscape for healthcare agents, based on a survey of recent literature. (a) Trend of publications, (b) frequency of top data modalities, (c) key technologies, and (d) application domains.

We present an exploratory trend analysis based on arXiv submissions retrieved using the keywords “healthcare agent” and “medical agent”, summarized in Fig. 2. The purpose of this figure is to illustrate general research activity patterns rather than provide an exhaustive bibliometric analysis. The counts reflect arXiv-indexed submissions as of Nov 2025 and include preprints. They do not correspond to the screened or included set of studies used in this survey. Results indicate that the publication volume has grown rapidly, with healthcare-focused agent research following the broader rise of AI agents since 2024. This growth reflects increasing research activity in the domain.

**Across modalities** (Fig. 2(b)), text-based inputs, including clinical notes and dialogue, remain dominant. EHR and medical imaging are also widely represented. Time-series data and genomics show a marked increase in 2025, indicating emerging interest in wearable sensing and precision medicine contexts. **Technically** (Fig. 2(c)), most work centers on agent frameworks, reasoning mechanisms, and multi-agent coordination. Recent growth is observed in knowledge graph integration and reinforcement learning, suggesting increasing emphasis on structured reasoning and adaptive decision strategies. **Application domains** (Fig. 2(d)) remain concentrated in general medicine, public health, and mental health. Drug discovery and genomics exhibit the strongest recent expansion, indicating a shift toward foundational biomedical research tasks.

Overall, the field demonstrates rapid expansion with increasing diversification in data modalities, technical methods, and application areas.

## 3. Survey methodology

**Fig. 3.**
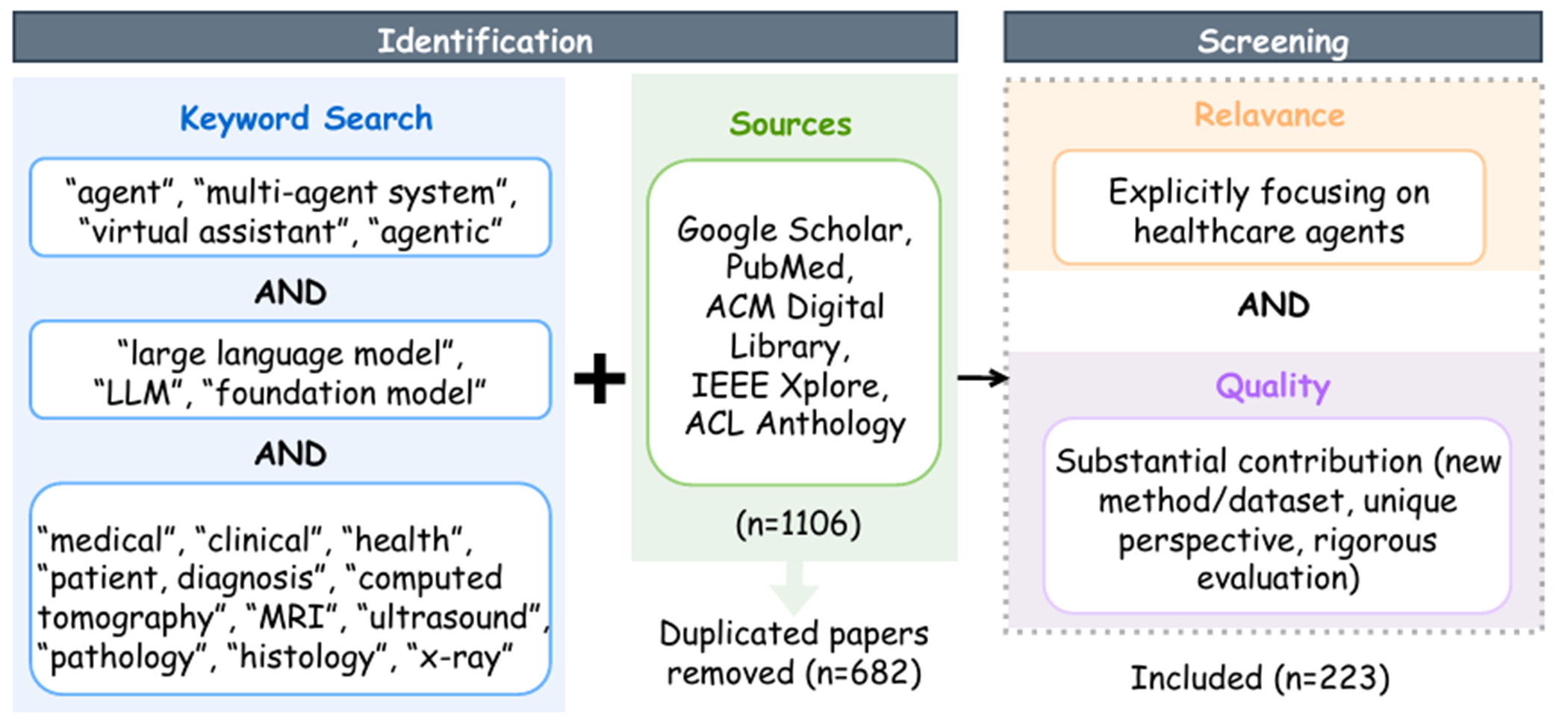
The PRISMA diagram illustrating the literature search process.

We conducted a systematic literature review following PRISMA guidelines (Fig. 3) to synthesize research on AI agents in healthcare. The goal was to provide a structured and reproducible overview using established evidence synthesis practices [26]. Searches were performed across five sources spanning artificial intelligence and medicine: Google Scholar, PubMed, ACM Digital Library, IEEE Xplore, and ACL Anthology. The final search was conducted on October 21, 2025. The Boolean query applied across databases was: (“agent” OR “multi-agent system” OR “virtual assistant” OR “agentic”) AND (“large language model” OR “LLM” OR “foundation model”) AND (”medical” OR “clinical” OR “health” OR “patient” OR “wellness” OR “diagnosis” OR “computed tomography” OR “X-ray” OR “MRI” OR “ultrasound” OR “pathology” OR “histology”). Given the rapid pace of development in this area, preprints were included alongside peer-reviewed publications.

### Inclusion and exclusion criteria.

Studies were required to meet two operational conditions. First, the primary objective needed to address a healthcare application, including diagnosis, triage, clinical documentation, treatment planning, patient interaction, or workflow support. Second, the system had to demonstrate explicit agentic behavior. A study was considered agentic if it reported at least one of the following: iterative control or decision loops, dynamic tool or resource invocation, maintenance of intermediate state or memory, multi-agent coordination, or planning followed by execution. We excluded systems limited to static prediction, single-pass generation, or question answering without action selection or iterative control. Work outside healthcare was also excluded.

### Screening process.

After finalizing the selection criteria, two authors independently screened titles and abstracts. Records advanced to full-text review if at least one reviewer judged them potentially relevant. Full texts were then examined by two authors, with disagreements resolved through discussion. Percent agreement on an overlapping subset prior to adjudication was 85%. The search returned 1106 records. After deduplication and preliminary filtering, 682 articles remained for detailed assessment, yielding 223 studies in the final corpus.

### Multi-label annotation procedure.

Annotation was carried out by seven authors using a predefined protocol. Before large-scale labeling, annotators jointly reviewed a pilot subset of papers (N = 10) to calibrate interpretation, refine category boundaries, and clarify borderline cases. This phase established shared expectations for applying the taxonomy. Given the size of the dataset, we adopted a primary–secondary model. Each paper was first labeled by one annotator and subsequently reviewed by a second author serving as an evaluator. Labels were derived from explicit descriptions in the manuscript. When discrepancies or uncertainties arose, cases were discussed among the team until consensus was reached. This workflow combined calibration, independent assignment, and structured verification, enabling consistent application of the taxonomy while remaining feasible at scale.

The label taxonomy spans technology (e.g., tool use, RAG), medical domain (e.g., radiology, genomics), task type (e.g., diagnosis, workflow automation), health stage (e.g., prevention, diagnosis), deployment location (e.g., home care, hospital), data modality (e.g., EHR, radiology images), evaluation paradigm, datasets, metrics, and reported challenges. Detailed label categories and the assigned labels for the included studies are available in a table within our open repository at link.

## 4. Perception and environment

The effectiveness of a healthcare agent depends on the data it perceives. In clinical contexts, input modalities such as natural language, EHR records, medical images, physiological signals, genomics, and audio define both the agent’s functional scope and its decision space. This section outlines how different modalities shape perception and action environments, and briefly discusses multimodal systems that integrate heterogeneous inputs. An overview of perception modalities, agent roles, and representative systems is provided in Table 2.

### 4.1. Single modality

#### 4.1.1. Natural language conversation

Natural language, particularly conversational interaction, is central to many healthcare agents. Dialogue unfolds over multiple turns and requires maintaining contextual continuity while interpreting patient-reported information. Agents operating in this modality support tasks such as symptom elicitation, triage, counseling, and collaborative deliberation. Recent systems illustrate different roles. Some agents conduct structured multi-turn questioning to refine diagnostic hypotheses [27]. Others simulate multidisciplinary discussion among virtual clinicians to support decision-making [28]. In mental health contexts, conversational agents guide structured therapeutic interactions [29].

##### Open Challenges.

Dialogue-based systems are often trained on static datasets and may not generalize to unpredictable real-world interactions [27]. Sustaining long-term coherence and producing clinically appropriate follow-up questions remain open problems that require domain-specific reasoning and validation.

#### 4.1.2. Electronic Health Records (EHRs)

EHR-based agents operate in structured data environments. Tasks involve translating clinical requests into executable queries over patient databases, such as identifying cohorts that meet specific diagnostic and laboratory criteria. Effective operation requires understanding database schemas and generating structured code, including SQL or Python. Systems such as EHRFlow [30] and TrustEHRAgent [31] illustrate this setting. EHRFlow uses multiple agents to decompose complex physician requests into smaller analytical tasks, while TrustEHRAgent acts as a verifier that estimates confidence in its outputs and abstains from uncertain predictions to maintain clinical safety.

##### Open Challenges.

Reliability remains a central concern, as errors in code generation can lead to incorrect cohort selection or unsafe conclusions [31]. Translating high-level clinical intent into precise executable queries is still difficult [30]. Privacy constraints further require secure local computation and strict data governance.

#### 4.1.3. Clinical notes

Clinical notes are textual records that capture longitudinal patient narratives. They shift the agent’s focus from real-time interaction to temporal extraction and synthesis. The agent must reconstruct a coherent clinical timeline from multiple time-stamped entries written by different clinicians over many years, identifying key events and assembling them into structured sequences. For example:

*[Time: −10y, Symptom: “forgetfulness”, Source: PCP]* → *[Time: −5y, Symptom: “getting lost”, Source: Neurology, Reporter: wife]* → *[Time: −2y, Symptom: “apathy”, Source: Psychiatry]*

This temporal reasoning defines the agent’s role as a diagnostic assistant integrating information across specialties. Infherno [32] transforms free-text notes into standardized FHIR resources, requiring both information extraction and validation against external terminologies such as SNOMED CT. CARE-AD [33] employs multiple specialized agents to analyze longitudinal clinical notes, each identifying domain-specific indicators of chronic conditions such as Alzheimer’s disease to produce an aggregated risk assessment.

##### Open Challenges.

Longitudinal reasoning remains difficult as early disease indicators are often sparse and distributed across years of documentation [33]. Ensuring adherence to complex output schemas such as FHIR also presents difficulties [32]. Additionally, clinical notes contain implicit knowledge and shorthand expressions, requiring contextual understanding for accurate interpretation.

#### 4.1.4. 2D medical imaging

Two-dimensional medical imaging introduces a visual modality that requires encoding pixel data into embeddings jointly processed with text. The nature of perception and reasoning varies by domain. In chest X-rays, the agent perceives a single global view and often acts as a radiology assistant. Systems such as MedRAX [34] combine planning with tool invocation, using classifiers, grounding models, or segmenters to localize abnormalities. In pathology, whole-slide images (WSIs) pose a different challenge due to their gigapixel scale. Perception becomes an active exploration process that mimics a pathologist’s workflow. Systems such as PathChat+ [35] and CPathAgent [36] employ a hierarchical structure where a supervisor agent inspects a low-resolution overview, generates hypotheses, and directs explorer agents to zoom into selected regions for detailed examination. The LLM coordinates these agents, integrating observations across magnifications until a diagnostic conclusion is reached.

##### Open Challenges.

In radiography, major issues involve fusing heterogeneous tool outputs and resolving conflicts among them. In pathology, efficient navigation of large images and multi-scale reasoning are central technical difficulties.

#### 4.1.5. 3D medical imaging

Three-dimensional imaging modalities such as Computed Tomography (CT) and Magnetic Resonance Imaging (MRI) extend the agent’s perceptual space from single images to volumetric structures. The agent may process these data either as unified 3D volumes or as sequential 2D slices, depending on the vision encoder and token compression strategy. In this environment, the agent functions as an anatomy-aware radiologist capable of understanding complex spatial relationships. CT-Agent [37] illustrates a modular approach in which specialized components are invoked for region-specific analysis, supporting structured report generation and targeted question answering. AgentMRI [38] focuses on quality control by detecting artifacts and selecting appropriate reconstruction methods. Other systems integrate volumetric features with clinical variables for downstream prediction tasks [39,49].

##### Open Challenges.

Processing large volumes introduces substantial computational costs due to the number of visual tokens. Capturing cross-slice spatial continuity remains difficult, as agents must recognize that findings across adjacent slices correspond to a single lesion rather than independent abnormalities. Anatomical variation often requires specialized modeling, and integration of volumetric interpretation with broader clinical context remains an open problem.

#### 4.1.6. Time-series signals

Physiological signals such as ECG, continuous glucose monitoring, and wearable sensor data capture dynamic temporal processes. Agents processing this modality translate qualitative user queries into structured analytical procedures. For example, when asked “Am I getting more fit recently?”, the agent interprets the query and formulates an analytical plan, as in the Personal Health Agent [40]: (1) filter activity data from the past three months, (2) compute average running speed per week, and (3) fit a regression model to detect trends. Translating natural language intent into a formal analysis plan is the core perceptual act.

Implementations differ in their operational focus. The Data Science Agent in PHA [40] executes analytical plans through code generation, while LLM-Powered Agent for PPG [41] focuses on signal diagnosis and tool selection for artifact correction.

##### Open Challenges.

Limited numerical reasoning capacity and token constraints hinder direct processing of long waveform data [41]. Interpreting metrics such as heart rate variability requires contextual grounding in personal and population baselines. Accurate signal diagnosis is also critical, as incorrect tool selection can compromise downstream analysis.

#### 4.1.7. Genomics & biomarkers

Genomic and biomarker data form a symbolic modality requiring knowledge-based reasoning rather than visual or conversational perception. Inputs such as gene sets, mutation lists (e.g., TP53, KRAS), or multi-omics profiles hold limited meaning without contextual information. The agent’s key perceptual act is to recognize biological entities and retrieve their functional relationships from external knowledge sources. Frameworks such as HEAL-KGGen [42] and GeneAgent [43] autonomously ground LLM outputs in curated biomedical resources by querying medical knowledge graphs or domain databases.

This environment situates the agent as a computational biologist automating bioinformatics workflows. AI-HOPE [44] converts natural language queries (e.g., “Compare survival outcomes for FOLFOX-treated patients with and without KRAS mutations”) into executable code that performs statistical analysis and generates survival plots, while HEAL-KGGen routes tasks to specialized agents [42]. GeneAgent [43] further introduces self-verification, proposing biological hypotheses and validating them against public databases to reduce factual errors.

##### Open Challenges.

A major challenge is factual hallucination, where an agent may incorrectly assert gene–disease links. Self-verification mechanisms, as in GeneAgent, directly mitigate this risk. Knowledge integration also remains difficult, requiring consistent fusion of LLM-based reasoning with structured biomedical graphs and multi-omics data. Finally, translating high-level clinical questions into precise analytical pipelines continues to require domain-specific reasoning.

#### 4.1.8. Audio

Audio adds temporal and affective information to the agent’s inputs. Perception proceeds in two stages. Systems such as ANNA [45] and automated survey agents [46] rely on transcription for language understanding, while prior work incorporates speech emotion recognition to infer affective states from prosody and pitch [50]. This dual pathway supports empathetic and diagnostic interaction. In therapeutic settings, the agent adapts responses to both content and detected affect, for example, shifting to supportive guidance when sadness is inferred.

##### Open Challenges

Transcription accuracy directly affect downstream reasoning [46]. Emotion cues are subjective and culturally variable, which limits reliability [50]. Subtle diagnostic markers in speech are easily confounded by noise, fatigue, and recording conditions, demanding robust models for real-world clinical use [45].

### 4.2. Multi-modality

Multimodality represents the most advanced stage of healthcare agents, where perception integrate heterogeneous inputs, including images, text, physiological signals, and genomic data, to form unified patient representations. Effective operation requires aligning cross-modal evidence and maintaining semantic consistency across data types. For example, genomic findings may need to be interpreted in conjunction with imaging or pathology results.

In this setting, the agent functions as a coordinator within a virtual multidisciplinary team. Frameworks such as MAM [47] and MedAgent-Pro [48] decompose diagnostic reasoning into specialized roles. In MAM, a generalist triages cases and routes data to domain-specific agents, while a director synthesizes their outputs into a final decision. MMedAgent [5] operates as a tool orchestrator that selects appropriate models based on the modalities involved—such as invoking a segmentation tool for images or a report generator for text. This coordination enables integrated decision-making beyond any single modality.

#### Open Challenges.

Key challenges include evidence fusion and conflict resolution, such as reconciling contradictions between textual and visual findings. Semantic alignment across symbolic and perceptual data is technically demanding. Progress is further limited by the scarcity of comprehensive multimodal datasets linking imaging, molecular data, and clinical outcomes.

## 5. Agent architecture and capabilities

Compared with single-pass LLMs, agentic systems introduce grounding and control loops that support more reliable and auditable behavior. We organize their core components by their typical order in a healthcare agent’s workflow. The process begins with **Knowledge Retrieval**, which provides verifiable evidence from medical literature and databases. We distinguish this from tool Use, which executes external computational utilities (e.g., calculators, segmenters), while retrieval focuses on acquiring knowledge. **Memory** complements retrieval by supplying persistent information of patient-specific details, maintaining continuity, and stabilizing reasoning. Together, these inputs support **Planning and Reasoning**, where the agent decomposes goals, forms hypotheses, and generates rationales. **Tool Use** then executes these plans using external resources. Finally, **Simulation** provides controlled environments for evaluation, while **Multi-Agent Collaboration** assigns specialist roles for coordinated decision-making. Table 3 summarizes these elements and representative systems.

### 5.1. Knowledge retrieval

Explicit knowledge retrieval is a critical advance over single-pass LLMs, allowing for the risk-aware and trustworthy behavior required in healthcare. Retrieval-augmented generation (RAG) is the most common grounding approach. Knowledge graphs (KGs) strengthen RAG through constraint-guided generation, and also support post-generation verification, improving risk awareness. In practice, systems typically use either Knowledge Graph (KG)-anchored RAG or text-anchored RAG. We describe these variants and how retrieval supports knowledge-based verification.

#### 5.1.1. KG-anchored RAG

KGs strengthen RAG by enabling entity canonicalization, relation-aware retrieval, and constraint-aware generation. Such systems map user queries to canonical biomedical entities and traverse the graph to assemble evidence. For example, AMG-RAG [51] and RUGGED [64] automate KG construction, couple graph traversal with external retrieval, reducing hallucinations and improving accuracy. MedGraphRAG [65] extends GraphRAG [66] with a triple graph over entities, papers, and lexicon definitions, while ClinicalRAG [67] further integrates multiagent systems to retrieve from heterogeneous medical knowledge. While KG anchoring improves disambiguation and safety checks, KG curation remains costly and may lag behind evolving clinical knowledge.

#### 5.1.2. Other RAG

Text- and tool-anchored RAG retrieve directly from authoritative sources, without explicit KG construction. Common patterns include query rewriting, dense/sparse retrieval, and evidence-conditioned generation. AI-VaxGuide [53] organizes a knowledge base of vaccination protocols and delivers context-sensitive guidance. An LLM-based CDSS [68] applies RAG across specialties for medication safety, while MALADE [69] is a pharmacovigilance pipeline that orchestrates agents over literature and regulatory interfaces. Collaborative frameworks like CLADD [70] retrieve from biomedical bases to contextualize molecules. Additionally, end-to-end training optimizes retrieval and reasoning policies, as shown by Deep-DxSearch [71]. Although lacking graphlevel constraints for multi-hop consistency and cross-source integration, these methods excel at flexible, rapid adaptation to evolving sources.

##### Knowledge-based verification

Beyond generation, systems can also verify and revise outputs using retrieved text or accumulated experience. MDTeamGPT [72] and ReflecTool [73] maintain long-term vector memories of validated answers or successful tool-use trajectories, retrieving them to steer subsequent rounds and apply LLM-based verification. While lacking formal rule-level guarantees, these knowledge-based verifiers significantly improve system safety and traceability.

### 5.2. Memory

Memory enables agents to maintain consistency and personalization across multiple clinical interactions. Beyond preserving past dialogues, memory modules coordinate domain knowledge, retrieve external resources, and adjust communication styles. Memory can be categorized into episodic, semantic, and demonstration memory.

#### 5.2.1. Episodic memory: Capturing specific interactions and context

Episodic memory forms the backbone of long-horizon agents by recording the *what, when, and where* of specific interactions, overcoming standard context window limitations to maintain continuity [74,75]. In practice, maintaining long-term memory in health chatbots fosters intimacy and encourages patients to share health information [76]. Similarly, in diagnostic simulations for mental health, AMC [54] samples critical information from dialogue history and medical records, and has achieved substantially higher accuracy. While beneficial for personalization, episodic memory requires careful management of privacy and scope.

#### 5.2.2. Semantic and knowledge-centric memory

Semantic memory focuses on organizing general facts, concepts, and relations, often leveraging structured formats like knowledge graphs to make agent reasoning more interpretable. By building a causal knowledge graph of a patient’s life events, an agent can generate transparent and personalized medical advice that is grounded in clear reasoning pathways [55]. Hybrid frameworks increasingly integrate structured semantic knowledge with episodic interaction histories, dynamically adapting agent behavior to align with user context and preferences [77]. This synergy enables agents to be both knowledgeable and personally attuned.

#### 5.2.3. Long-term and demonstration memory

Another approach involves using long-term memory to store a library of successful past experiences or demonstrations that guide future decisions. In the context of reasoning over electronic health records, an agent can improve its planning by dynamically retrieving relevant past cases from memory to inform its current task [6]. This principle also applies to complex medical tool use, where an agent can learn from a repository of successful problem-solving trajectories to select the right tool and correct its actions [73]. At scale, central memory hubs serve multiple specialized agents by integrating accumulated experience with current medical knowledge to effectively navigate complex scenarios like rare disease diagnosis [78].

### 5.3. Planning and reasoning

In clinical settings, planning denotes the decomposition of tasks such as diagnosis, therapy selection, and follow-up into actionable steps. Reasoning denotes the analysis of evidence, the application of domain knowledge, and the formation of testable hypotheses. Challenges include high stakes and uncertainty, incomplete or noisy inputs, evolving patient states and literature, and requirements for transparent justification. Recent systems address these challenges by embedding explicit reasoning and planning within single- and multi-agent frameworks [79].

#### 5.3.1. Planning and task decomposition frameworks

Effective agents factor long-term clinical objectives into tractable sub-tasks and reusable procedures, performing **goal decomposition**. For instance, STELLA [56] treats planning as a reusable template library, while simulated-patient systems [80] use multi-agent roles with knowledge retrieval to coordinate summarization, QA, and evaluation. Operational planners extend the same principles beyond diagnosis: MedScrubCrew [81] formulates appointment scheduling as constrained matching and integrates a knowledge graph with agent policies to align patient and provider profiles. Modality-specific decompositions follow naturally; CT-Agents [37] partitions volumetric interpretation by anatomy region with compact representation. Long-term memory modules further link plans across encounters, encouraging continuity and stable defaults over time [76]. Taken together, these systems frame planning as program-like composition, with templates, constraints, and memory supporting reuse and adaptation.

##### Action-oriented planning and reasoning.

A complementary design interleaves planning with tool execution so that observations revise subsequent steps. For example, MeNTi [57] nests tool calls for clinical computations, and MMedAgent [5] trains agents to choose modality-specific tools. In procedural domains, SurgBox [82] combines retrieval with a surgical copilot and a long–short memory mechanism to balance immediate assistance with persistent knowledge. Graph-aligned approaches follow the same action–reflection loop: KGARevion and KERAP interleave reasoning with calls to knowledge graphs and calculators under a ReAct-style scheme [83,84]. Domain-focused agents such as GeneAgent [43] act on retrieved gene-set statistics and revise explanations after verification. Across these designs, planning is tightly coupled to execution, with tool feedback and verification signals guiding iterative refinement.

#### 5.3.2. Reasoning: Enhancing decision depth and reliability

**Sequential reasoning** models emulate a clinician’s step-by-step thought process. Chain-of-Diagnosis (CoD) [85] converts diagnostic inference into a stepwise program and produces a calibrated disease distribution. Role-structured multi-agent dialogues [79] assign complementary responsibilities to reduce bias and expose assumptions. CareCall [76] further utilizes long-term memory modules to support continuity across encounters, though such a design raises privacy and governance considerations.

##### Exploratory and structured reasoning.

More complex medical problems require exploring multiple reasoning paths. Graph-based approaches address this by expanding hypotheses and consolidating evidence across branches, as tree-of-reasoning [86] does with differential diagnoses. For biomedical question-answering, ESCARGOT [87] organizes thoughts as a dynamic graph aligned with a knowledge graph to reduce unsupported claims and improve transparency. For radiology, CT-Agent [37] dispatches specific reasoning paths for volumetric analysis, and STELLA [56] augments reasoning with an evolving template library and a dynamic tool ocean. Graph-anchored retrieval frameworks such as AMG-RAG and Medical Graph RAG integrate reasoning with knowledge-graph traversal for evidence alignment [51,65].

##### Reflective and self-correcting reasoning.

Safety-critical healthcare applications require agents that can critique and revise their outputs. One line of work verifies claims against trusted resources: GeneAgent [43] queries domain databases to validate gene-function outputs, and KGARevion [83] extracts latent triplets and checks them against a knowledge graph. A second line integrates verification into the reasoning process via coordinated roles: KERAP [84] aligns linkage, retrieval, and prediction agents on a shared graph to produce calibrated outputs. A third line emphasizes continual improvement: RAG-KG-IL [88] combines retrieval with incremental updates to the knowledge base to reduce hallucination over time. Complementarily, evaluation-driven reflection closes the loop: GEMA-Score [89] parses generated radiology reports and provides explanatory feedback, while AutoCT [90] applies search-based refinement to iteratively improve feature sets and decisions. Together, these approaches formalize self-critique and revision as explicit steps, improving reliability and traceability.

### 5.4. Tool use

In clinical settings, where text-only responses are insufficient, agents must invoke external tools to execute multi-step tasks, transforming plans into verifiable procedures and yielding three practical benefits: improved safety and reliability through grounded computations with full traceability [53,91,92]; stronger explainability and accountability via auditable call logs, provenance tags, and intermediate results [93]; and higher productivity by automating routine steps such as note pre-population, templated synthesis, guideline-concordant ordering, and scheduling [53,92,94–96].

#### 5.4.1. Types of tool use in healthcare

**Retrieval and knowledge tools** sit upstream and ground reasoning in external evidence. Classical RAG systems can query medical literature (PubMed, clinical guidelines), patient EHRs, and specialized databases to retrieve related evidence [53,91]. STRID [91] further introduces an advanced implementations orchestrate multiple retrieval strategies, combining dense retrieval for semantic similarity and sparse retrieval for keyword matching. Moreover, domain-specific retrieval extends beyond literature: genomic agents query variant databases (ClinVar, gnomAD) [43,97], drug discovery agents access compound libraries [58,98], and diagnostic agents retrieve similar cases from institutional repositories [93]. **Computational tools** operate midstream by transforming inputs into quantitative estimates that can drive decisions. Risk stratification agents employ validated calculators (Framingham, ASCVD) [92], radiotherapy agents interface with dose optimization algorithms [99], and genomic agents invoke sequence aligners and variant effect predictors [43,97]. These tools provide numerical outputs with established clinical interpretation thresholds, reducing recommendation ambiguity. **Workflow integration tools** enact decisions downstream and connect agents to care delivery. FHIR APIs [32,94] allow agents to read structured clinical data and write orders, care plans, and documentation. Systems like IMAS [100] orchestrate triage workflows, while conversational agents [96] pre-populate intake forms. Privacy-preserving architectures implement role-based access control, ensuring minimal necessary privileges [94]. Orthogonally, **simulation and verification tools** ensure reliability before deployment. Platforms like AgentClinic [101] provide simulated patient encounters, while verification tools check internal consistency [43] and cross-validate against clinical guidelines [53].

#### 5.4.2. Agentic progression

Agent sophistication is measured by the ability to *select, sequence, and adapt* tool use as contexts evolve, marking a shift from scripted automation to genuine autonomy. At the simplest end, **Single-step invocation** is a deterministic tool use in which an input is mapped to a single tool; systems for structured clinical assessments [45] and radiology report generation [93] often trigger terminology services in this way, which is effective for narrowly defined tasks yet limited when the first call leaves uncertainty. Moving beyond this, **Sequential workflows** chain tools in predefined sequences: genomic interpretation agents [43,97] progress from variant calling and filtering to prediction, association search, and report generation, while radiotherapy planning [99] iteratively coordinates dose calculation, complication modeling, and quality evaluation; execution continues until sufficient certainty is reached. Further, **Dynamic selection with reasoning** chooses which tools to invoke based on the evolving clinical context; multi-agent diagnostic systems [78,102] switch among genetic databases, metabolic analyzers, and case repositories as hypotheses shift, and AgentMD [59] learns tool-selection policies from EHR data, prioritizing calculators and tests that historically provide the highest diagnostic value for a given presentation, consistent with pretest-probability reasoning. At a higher level, **Self-verification and adaptive orchestration** implements closed-loop control: GeneAgent [43] automatically invokes verification tools, detects inconsistencies, retrieves additional evidence, and revises conclusions, reporting a 40% reduction in hallucinations; rare-disease agents [78] maintain competing hypotheses with parallel tool invocations and adjudicate among them, and error-recovery mechanisms detect tool failures and retry with alternatives. Finally, **Meta-learning and discovery** captures systems that learn which tools work best in particular scenarios and synthesize new combinations: BioScientistAgent [103] uses reinforcement learning to discover effective drug-repurposing query sequences, outperforming hand-designed workflows; more generally, agents may improve the toolkit itself.

### 5.5. Simulation

Persona design is a core technical component for ensuring that AI agents in healthcare are controllable, trustworthy, and effective. A well-designed persona aligns an agent’s behavior with clinical norms and user expectations, a critical requirement in high-empathy domains. As LLM agents expand in healthcare, persona-driven conditioning is essential for maintaining system alignment and safety [104]. This section dissects this technical lifecycle, from instantiation and regulation to validation and frontier challenges.

#### 5.5.1. Persona instantiation and regulation

Persona instantiation translates an abstract clinical role into a computational representation, often drawing from established practices like Cognitive Behavioral Theory [29]. These personas, such as patients or providers, are already used to improve clinical communication in applications like Talk2Care [60]. Technically, this is achieved through explicit and parametric encoding. Explicit encoding via prompting is the most flexible method, ranging from simple prompts that define a character’s traits [105] to more advanced approaches such as Behavioral Tokenization, which uses special tokens for fine-grained behavioral control in BehaviorSFT [61]. Other methods, such as LAPI [106], use objective-constrained prompting to align responses with a professional identity.

However, a well-defined persona is effective only if it is regulated during dynamic interactions. To maintain consistency and combat ‘‘persona drift’’, a risk highlighted by memory failures in commercial chatbots [107], systems require robust memory architectures, as discussed in AnnaAgent [108]. Beyond consistency, effective regulation requires dynamic adaptation, typically achieved by modeling the user’s state to inform the agent’s strategy. This allows agents to selectively apply clinical techniques like Motivational Interviewing based on user progress [109] or calibrate their feedback style to a clinician’s expertise level [110].

#### 5.5.2. Persona validation and frontier challenges

Validating a persona requires a multidimensional protocol spanning technical fidelity, user perception, and task performance. On the technical axis, benchmarks quantify behavioral fidelity against predefined strategies [61]. On the human axis, granting users control over persona configuration improves perceived trust and engagement [111]. These considerations should connect to task outcomes: for example, Medco [62] employs role-specific agentic copilots to deliver training simulations for medical students, while VChatter [112] adopts therapeutic roles to support exposure-based interventions.

First, a persona generalization gap persists: personas validated in one cultural context may fail in another, as generative agent societies can diverge from real-world public health attitudes [113]. This gap elevates risks of bias, manipulation, and harm, motivating stronger evaluation frameworks and risk assessments [107,114,115]. Second, system-level complexity complicates multi-agent deployments: coordinating heterogeneous personas for composite reasoning remains open, including in role-playing expert systems [28]. Third, embodied interfaces introduce consistency requirements between verbal and non-verbal behavior [116]. Across these settings, practical deployments continue to benefit from human oversight to enforce persona boundaries and safety constraints [117].

### 5.6. Multi-agent systems

LLM-based multi-agent systems (MAS) provide an orchestration layer over the primitives outlined above. By assigning specialist roles and regulating communication, MAS enable task decomposition, cross-checking, and consensus formation. Coordination typically uses role specifications, shared state or memory, and judge or mediator agents that reconcile divergent hypotheses while preserving provenance. This paradigm is useful across diverse domains, from multimodal cardiology diagnostics [118] and debate-based mental health counseling [119] to synthetic medical dialogue generation [120]. Comparative studies indicate advantages over single-agent baselines on complex tasks that benefit from complementary expertise and structured disagreement resolution [121]. In practice, MAS integrates knowledge grounding, planning, and tool use within each role and exchanges intermediate results through constrained protocols, improving robustness and transparency in workflows that mirror team-based clinical practice.

#### 5.6.1. Architectural patterns & organizational structures

Multi-agent architectures in healthcare mainly differ by their organizational structure and distribution of decision authority, falling into hierarchical/centralized, flat/decentralized, and hybrid patterns.

##### Hierarchical & Centralized Architectures

This structure mirrors clinical team dynamics: a ‘‘manager’’ or ‘‘director’’ agent assigns specialized diagnostic tasks in fields such as cardiology, forensic pathology, and general medicine, then aggregates the results into a final report [47,118,122]. This pattern is also widely adopted to ensure safety and guideline adherence. For instance, systems often implement tiered oversight where junior agents propose actions, mid-tier agents check for harm, and a senior agent, or a human doctor, provides final approval [72,123,124]. Beyond clinical reasoning, centralized architectures orchestrate complex AI pipelines, with a controller coordinating agents dedicated to data handling and model training [125], or a meta-agent synthesizing information retrieved by multiple agents from EHRs [126]. These designs ease coordination and align with institutional oversight. Risks include single-point failure, reduced diversity of perspectives, and sensitivity to errors at the coordinator.

##### Flat & Decentralized Architectures

Flat architectures empower agents with equal status to engage in peer-to-peer debate or voting. This approach is frequently used to tackle complex clinical reasoning, where multiple specialist agents debate evidence to identify conditions, disambiguate similar diseases, or provide empathetic counseling [102, 119,127,128]. The same peer-review structure is effective in multi-modal reasoning, such as radiology VQA, where context, reasoning, and verification agents collaborate to finalize an answer [129]. Beyond diagnostics, decentralized debates can enhance research creativity through continuous knowledge exchange [130] or improve robustness by intentionally injecting dissent to overcome silent agreement bias in group discussions [131]. Although these frameworks promote viewpoint diversity and resilience, they require sophisticated consensus mechanisms.

##### Hybrid Architectures.

Hybrid designs blend hierarchical coordination with peer-to-peer collaboration to balance efficiency and diversity. Some systems create specialized peer groups for tasks like data extraction and captioning, which are then overseen by a higher-level filtering or review agent [132,133]. Others combine different functional agents, such as those for retrieval, knowledge graph integration, and scoring, to produce more reliable and explainable outputs [88,89]. A third pattern uses hybrid structures to simulate cognitive modularity, where distinct agents handle analysis, synthesis, and validation in a coordinated workflow, or engage in an ‘‘inner dialogue’’ to guide users. These approaches have shown significant accuracy improvements in complex domains like neurological problem-solving [86,134,135]. Hybrids thus capture both the coordination advantages of hierarchies and the creative diversity of decentralized debates.

#### 5.6.2. Coordination & communication mechanisms

**Fig. 4.**
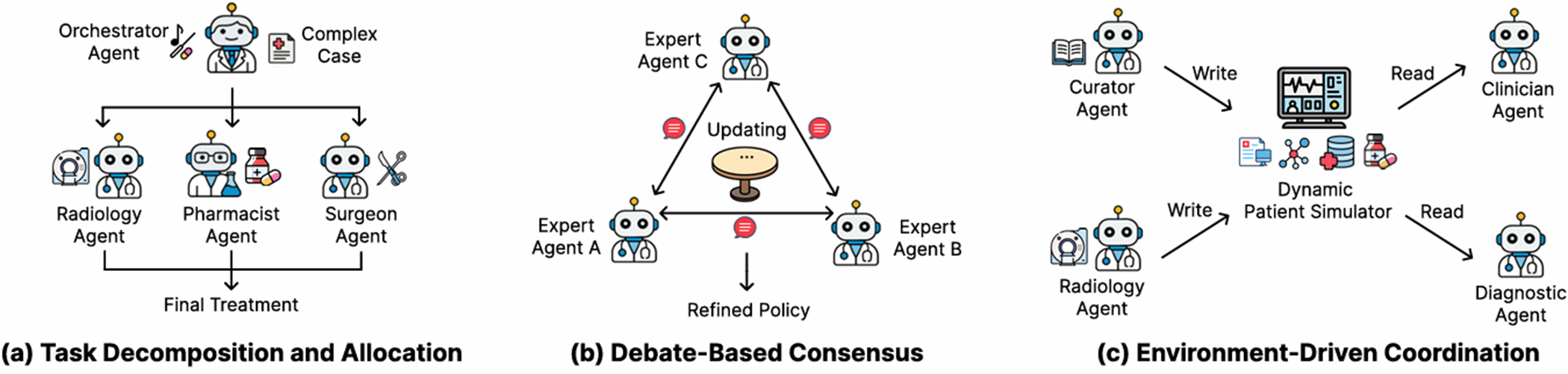
Three coordination mechanisms for healthcare multi-agent system: (a) Task decomposition and allocation via an orchestrator assigning sub-tasks. (b) Debate-based consensus among peer agents. (c) Environment-driven coordination through a shared, persistent environment (e.g., a patient simulator).

Coordination is how multi-agent systems decompose complex tasks, interact, and converge on solutions. As shown in Fig. 4, three dominant mechanisms appear: (a) task decomposition and allocation, (b) debate-based consensus, and (c) environment-driven coordination.

##### Task Decomposition & Allocation.

Hierarchical MAS naturally excel at decomposing complex workflows into manageable subtasks. This is common in diagnostic pipelines, where a primary agent splits a case into sub-problems for specialist agents (e.g., GP, radiologist) and integrates their findings, as in MAC [63], MAP [123], MAM [47], and FEAT [122]. The approach also extends to automating complex technical and analytical workflows, such as configuring machine learning pipelines [125], generating descriptions for pathology images [136], or performing thematic analysis of interview transcripts [137]. More advanced systems introduce dynamic team formation, where specialist agents can join or leave the collaboration based on evolving information needs, enabling a more adaptive diagnostic process [138].

##### Debate-Based Consensus.

Debate mechanisms leverage the argumentative and reasoning capabilities of LLMs to refine solutions. In clinical settings, structured debates help disambiguate similar diseases, formulate empathetic counseling responses, or synthesize evidence from data-driven and knowledge-driven perspectives [102,119, 128]. A key benefit of this adversarial process is the mitigation of biases and hallucinations. By encouraging agents to challenge each other, systems can avoid premature convergence and surface conflicting evidence [127]. Some frameworks explicitly introduce a ‘‘dissenter’’ agent to disrupt groupthink [131], while others use dedicated validation agents to cross-examine diagnoses, both leading to more robust and accurate outcomes [129,134].

##### Environment-Driven Coordination.

Many MAS coordinate asynchronously by reading and writing to a shared environment that persists state and mirrors clinical workflows. One line of work employs dynamic patient simulators: agents conduct note-grounded dialogues with synthetic patients [120] and query MIMIC-based simulators for vitals and labs, updating a shared case representation over time [138]. A second line uses shared knowledge structures, such as PathChat+ [35], so digital pathology agents log interim report findings to refine diagnoses collaboratively, while curator agents like RAG-KG-IL [88] incrementally add literature-derived facts to a central KG that others use as a stable context to curb hallucinations. Finally, existing structured records can serve as the environment: agents extract features from radiology reports [89], retrieve EHR histories to condition recommendations [126], and scan imaging repositories to surface confounders for association studies [39]. Collectively, these designs replace direct messaging with a persistent, queryable state that supports reproducible multi-agent clinical workflows.

#### 5.6.3. Key technical challenges & future directions

Despite impressive progress, LLM-based MAS face several open challenges. **Shared Context & State Consistency** remains central: compared with ad-hoc histories, structured state externalization and persistent knowledge graphs reduce contradictions and hallucinations [86, 88,127,134]. **Communication Overhead & Protocol Design** calls for constrained interfaces—well-defined APIs, concise list-based messages, and tiered pipelines—to control cost and error propagation [124,125, 136]; in parallel, dynamic team formation improves efficiency [138]. **System-Level Evaluation & Alignment** requires end-to-end metrics because component-level gains can degrade overall performance [121]; useful signals include expert-correlated scoring and guideline concordance across datasets [47,89,123], with layered oversight and deliberation to detect harms and mitigate bias [124]. **Agent Frameworks & Scalability** benefit from modular designs and benchmarked team composition [125,136,138], yet long-horizon coordination and history management introduce overhead in clinician-in-the-loop settings [72, 137,139]; advancing scheduling, memory, and resource allocation remains a key direction.

Future work must focus on developing persistent memory for consistent patient context, designing efficient communication protocols, and devising comprehensive, system-level evaluation metrics that account for safety and ethics. Emerging directions like dynamic team formation [138], deeper integration of knowledge graphs [88], and fairness-aware deliberation [124,139] are promising. By addressing these challenges, LLM-based MAS are poised to become integral partners in the future of healthcare delivery.

## 6. Applications across the healthcare ecosystem

This section reviews practical applications of AI agents across the healthcare ecosystem. Unlike the preceding technical taxonomy, it is organized around the primary stakeholders these systems serve. This role-based view helps clinicians, patients, researchers, educators, and administrators identify relevant developments and understand how agent roles, autonomy, and human oversight differ across contexts. Table 5 summarizes the main application areas of healthcare agents, including typical autonomy levels, describing their typical roles, core capabilities and representative systems across stakeholders. To make oversight requirements explicit across application areas, we introduce a pragmatic four-level risk tiering (R0–R3) based on the potential severity and directness of harm if an agent is incorrect.

- **R0 (Administrative and operational):** Errors primarily cause workflow, compliance, privacy, or logistical harm, with low direct patient safety impact.
- **R1 (Patient-facing informational):** Errors may misinform patients or degrade self-management, without directly driving clinical decisions or actions.
- **R2 (Clinical decision support):** Errors may influence diagnosis, triage, or treatment planning, with potential downstream clinical consequences even under clinician sign-off.
- **R3 (Direct/time-critical):** Errors may trigger immediate harm through direct interventions or time-sensitive actions in clinical workflows.

This tiering is an operational synthesis for organizing oversight requirements rather than a regulatory classification, and the assigned tier reflects the highest plausible harm in the intended workflow. Table 4 operationalizes this autonomy–risk view by summarizing suggested minimum oversight and controls across autonomy levels and risk tiers. Notably, across the reviewed systems we did not identify fully autonomous A3 deployments, consistent with the safety-critical nature of clinical settings and prevailing expectations for human oversight.

### 6.1. Supporting doctors: Enhancing clinical workflow

Doctors are key decision-makers in complex clinical environments. For them, AI agents evolve from passive tools to active collaborators. These systems assist with adaptive decision-making by integrating distributed data and coordinating tasks. Their goal is to enhance clinical judgment while preserving human oversight. This section follows a typical workflow: diagnosis and decision support, documentation, and workflow automation.

#### 6.1.1. Diagnosis & decision support

Clinical diagnosis relies on integrating patient data with medical knowledge. Recent agentic systems move beyond information retrieval and image analysis, treating each module as a callable tool to enable higher-level planning and reasoning within clinical workflows. This section outlines three directions: (1) constructing reliable informational foundations for diagnosis, (2) supporting diagnostic reasoning, and (3) using simulation to model patients or clinicians in diagnostic tasks.

##### Building the Informational Foundation.

A core challenge in diagnosis is extracting accurate and relevant insights from fragmented clinical data. Agents address this by transforming unstructured inputs into standardized and interpretable representations such as FHIR resources [32]. They extract key variables like Gleason scores from pathology notes [147] or psychosocial factors from interviews [148] to create structured patient profiles.

To make these profiles trustworthy, agents must keep their outputs aligned with the continually evolving body of medical knowledge. For example, AI-VaxGuide [53] integrates RAG with immunization guidelines, while others ground recommendations in standards like the American Diabetes Association [149]. To reduce hallucination, systems like GeneAgent [43] incorporate self-verification loops against authoritative databases. Using external tools further enhances agents’ capacity to produce evidence-grounded insights. AgentMD [59] converts free-text notes into risk scores using calculators, while TxAgent [140] orchestrates over 200 resources to generate personalized treatment plans. In oncology, agents combine vision models and guideline retrieval to improve diagnostic accuracy [150]. Together, these systems illustrate how agents convert raw data into structured, evidence-grounded recommendations, establishing a dependable foundation for clinical decision support [5,34,151].

##### Diagnostic Reasoning.

Beyond data preparation, agents are increasingly applied to diagnostic reasoning. They adapt analytical strategies across modalities and coordinate information to support accurate and explainable decisions.

In pathology, agents analyze whole-slide images at multiple magnifications and distribute tasks among specialized modules to improve cancer classification and generate visually grounded reports [35,36, 136]. Large-scale foundation models trained on millions of slides further enable zero-shot classification and biomarker prediction [152]. In radiology, CT-Agent [37] decomposes 3D volumes into anatomical subtasks for visual question answering, while AT-CXR [153] introduces adaptive triage that determines when to automate and when to defer to radiologists under uncertainty.

Beyond imaging, agents interpret genomic results into guideline-based recommendations [42,44,154] and use longitudinal health records to forecast disease trajectories [33,93,155]. Knowledge graphs further enhance reasoning by encoding links among symptoms, diseases, and treatments. They enable multi-step reasoning across biological entities, combine evidence from multiple sources, and integrate diverse data types to achieve diagnostic accuracy comparable to human experts [84,118,156].

##### Clinical Simulation.

A key strength of AI agents is their ability to simulate interactions in complex healthcare environments. *Patient-centered simulations* create digital twins from longitudinal records, enabling systems like EHR2Path [157] to forecast hospital trajectories instead of predicting isolated outcomes. Organ-Agents [158] extends this by modeling interconnected physiological subsystems, allowing clinicians to test counterfactual scenarios (e.g., delaying treatment), supporting reasoning in high-risk settings [159].

*Doctor-centered simulations* replicate collaboration by coordinating virtual specialists who deliberate and converge on treatment strategies, achieving diagnostic accuracy comparable to human teams [128, 134]. Platforms such as ColaCare [126] coordinate specialist agents to reach consensus and improve safety, while frameworks like Dynami-Care [138] dynamically recruit them based on case-specific needs. ClinicalLab [160], on the other hand, establishes standardized benchmarks for evaluation. Together, these systems demonstrate how simulation integrates reasoning and collaboration into a cost-efficient and safe approach to clinical decision support.

#### 6.1.2. Clinical documentation & reporting

Clinical documentation and reporting convert computational outputs into medico-legal records that must remain faithful to source data and verifiable against evidence. Early neural systems for radiology reporting [161] and conversational summaries [162] showed feasibility but lacked transparency and verification. These limitations motivated agentic approaches, which decompose documentation into modular, evidence-based workflows to ensure traceability and auditability. This shift is illustrated in the following two domains.

##### Structuring Clinical Interactions.

Encounter notes are a natural entry point where the main challenge is turning unstructured, multi-party conversations into structured, actionable information. Agents generate context-aware representations that integrate external knowledge and reasoning [163,164], while some convert text directly into interoperable formats, such as Infherno [32], which embeds FHIR resources into documentation.

In real-world use, Mo was deployed with over 900 patients and improved clarity and physician satisfaction, with 95% of ratings classified as good/excellent [165]. Similar pipelines are also applied outside of documentation. FRAME [141], for instance, produces scientific manuscripts with quality comparable to human authors.

##### Grounding Reports in Visual Evidence.

Another direction centers on generating formal reports from multimodal data, where each claim must be supported by evidence. Agents replicate the stepwise workflow of human experts, introducing checkpoints and tool integration for verification. In radiology, multi-agent ‘‘councils’’ distribute tasks for retrieval, image analysis, drafting, and review, improving accuracy and reducing hallucinations [133,166]. Pathology adopts similar strategies at gigapixel scale. PathChat+ [35] uses hierarchical pipelines to navigate whole-slide images at multiple magnifications, keeping diagnostic reports visually grounded.

#### 6.1.3. Workflow automation

Clinical documentation records both patient states and clinician actions, forming a foundation for workflow automation. Building on this, intelligent agents streamline practice across three levels: data management, clinical knowledge application, and support for human interaction.

##### Data Access and Organization.

At the data level, agents provide natural-language interfaces to complex healthcare databases, simplifying querying and retrieval. EHRFlow [30] generates privacy-preserving SQL and FHIR queries with iterative debugging. EHRAgent [6] treats multi-table reasoning as a tool-use planning task supported by a code interpreter. ClinicalAgent [167] retrieves comprehensive trial evidence (e.g., safety reports, enrollment data) to assess feasibility, and Talk2Bio models (T2B) [168] extends this idea to systems biology, enabling users to query and simulate biological models. Together, these systems reduce manual effort and error in retrieval, allowing clinicians to access longitudinal data through simple queries.

##### Application of Clinical Knowledge.

At the knowledge level, agents apply established medical rules and protocols, shifting from retrieval to rule-based execution. CDR-Agent [142] applies validated decision rules in emergency care to reduce unnecessary imaging. In radiotherapy and MRI reconstruction, planning agents automate workflows to optimize organ protection and image quality [169,170]. In public health, the Decision-Language Model (DLM) [171] encodes clinical and equity priorities into reward functions that guide intervention allocation. Across these domains, agents function as dependable executors of evidence-based procedures rather than independent decision-makers.

##### Support Human Interaction.

At the interface level, agents mediate interactions between clinicians, patients, and software systems. Web-based agents with visual perception automatically navigate EHR platforms for data entry [172], or translate technical radiology reports into patient-friendly language [172]. Higher-level platforms such as MedicalOS [173] translate natural-language instructions into software commands for retrieving records or managing examinations, reducing navigation effort. In surgical settings, systems like SurgBox [82] act as real-time copilots, coordinating information and assisting intraoperative decisions.

### 6.2. Empowering patients: Fostering engagement and wellness

Patients are increasingly active in managing their own health, creating a need for personalized and continuous support beyond the clinic. For this group, agentic systems provide scalable and on-demand engagement by using memory and persona conditioning to support care delivery. Direct interaction also introduces specific risks, particularly in areas such as mental health, where patient vulnerability and the consequences of inappropriate interventions require strong safeguards. This discussion is organized by intervention type and associated risk, beginning with therapeutic applications and then turning to supportive systems for patient education and self-management.

#### 6.2.1. Mental health and counseling

Mental health applications represent one of the most sensitive domains in healthcare. Agentic systems in this area span a spectrum, from tools that assist clinicians in assessment to therapeutic platforms that engage directly with patients. The latter requires robust safeguards given patient vulnerability, the possibility of crisis situations, and the serious consequences of inappropriate interventions.

##### Clinician Support for Assessment and Diagnosis.

Agentic systems are being developed as auxiliary tools for clinicians, primarily to structure complex assessment protocols and analyze diverse behavioral signals under professional oversight. Multi-agent frameworks can operationalize standardized psychiatric protocols into reliable computational workflows. A representative example is MAGI [144], which converts the MINI interview into a guided diagnostic process by specialized agents. Debate-based architectures extend this by using argumentation to clarify ambiguity [119]. Beyond structured interviews, systems have been proposed to extract diagnostic signals from varied sources, including patient narratives for identifying cognitive distortions [174], social media activity for detecting disorders [175], and facial expressions for recognizing emotional states [116]. Other efforts focus on non-verbal inputs, such as PsyDraw [176], analyzing children’s House-Tree-Person drawings with multi-agent feature extraction. Additional systems cluster around knowledge-graph reasoning for interpretable differential diagnoses [177], interactive assessment formats [178], and risk-focused tools such as MentalRAG, which monitors patient data to identify suicidal ideation and notify clinicians [179].

##### Patient-Facing Therapeutic Interventions.

When agentic systems shift from assisting clinicians to delivering therapy directly, the safety requirements become more stringent. Empathy and therapeutic alliance are central in this context. CAMI [109] guides conversations using motivational interviewing, while AutoCBT [143] delivers cognitive behavioral therapy through a multi-agent framework. Other applications target specific needs: VChatter [112] simulates social interactions for exposure therapy, while PDC30 [180] offers psychoeducational support for dementia caregivers. Safety is addressed through continuous monitoring, as in EmoAgent [181], and through escalation protocols embedded in conversational frameworks [163]. A further line of work explores multi-role counseling agents, exemplified by MIND [135], which distributes therapeutic responsibilities across different LLMs to emulate collaborative counseling teams.

##### Evaluation and Governance.

Given the high stakes of patient-facing systems, evaluation and governance are critical. Emerging taxonomies identify risks specific to AI-assisted psychotherapy, including threats to therapeutic alliance and failures in crisis management [115]. Benchmarks such as ESC-Judge [182] provide systematic assessments of empathy and therapeutic quality. Challenges remain, including disparities in model performance across demographic groups, which raise fairness concerns [183]. At the same time, offline-capable models [184] expand access by enabling support in low-connectivity environments. Ensuring that these systems augment rather than replace professional clinical judgment remains central to their responsible use.

#### 6.2.2. Patient education and self-management

AI agents are increasingly deployed as interactive health coaches that explain diseases, treatments, and prevention strategies to improve health literacy. A defining capability of these systems is personalization. They adapt language and cultural framing to individual profiles, using user-adjustable personas that align with clinical norms to foster trust and engagement [61,106,111]. When combined with empathic responses and autonomy-supportive phrasing, these systems help patients make informed decisions and adhere more consistently to care plans.

##### Evidence-Grounded and Personalized Education.

To provide reliable guidance, patient-facing agents rely on RAG to ground explanations in current knowledge rather than latent model memory. AI-VaxGuide [53] transforms vaccination guidelines into an interactive base for context-sensitive delivery. Long-term memory mechanisms allow agents to recall personal details and prior progress, strengthening trust [76], while planning capabilities enable adaptive, adaptive goal-setting for chronic condition management, such as incremental lifestyle adjustments or regular mental health check-ins. Together, these strategies illustrate how personalization, evidence-grounding, and longitudinal support converge in patient education.

##### Connected Self-Management and Care Coordination.

Agentic systems increasingly extend their role by linking self-management with clinical workflows and remote monitoring. Wearables and home devices provide real-time inputs. FHIR-aware multi-agent frameworks [94] allow privacy-preserving EHR integration, and patient narratives can be converted into structured FHIR resources for clinical use [32]. Beyond integration, agents automate follow-ups and collect structured status updates to support care coordination [96]. These capabilities show how patient education tools evolve into connected platforms that sustain self-management while ensuring timely links to professional care.

### 6.3. Advancing medical science and education

Researchers and educators are central to the healthcare ecosystem because they generate medical knowledge and train clinicians. Their work includes two major challenges: drawing hypotheses from large, heterogeneous biomedical data, and building scalable, interactive training environments. AI agents support both roles, as research accelerators for discovery workflows (e.g., genomics and drug development) and as simulators of patients or peers for clinical training. This section is organized around these two functions: knowledge creation in biomedical research and knowledge dissemination in medical education.

#### 6.3.1. Biomedical research and discovery

Biomedical research depends on systematic hypothesis formation, careful experimental planning, and the integration of diverse data and literature. The scale and complexity of genomic and proteomic data, high-resolution microscopy, and large biomedical text corpora pose specific challenges. Systems based on LLMs are beginning to assist clinicians and researchers by interpreting these data and linking them to decision-making processes [98].

##### Hypothesis Generation and Experimental Design.

A central role for agents in research is supporting scientific reasoning and hypothesis generation. Stella [56] identifies literature gaps and proposes new hypotheses with corresponding experiments. BioScientistAgent [103] applies a similar approach to drug discovery by integrating evidence for drug repurposing and suggesting testable interventions. DrugAgent [58] further extends this paradigm by translating high-level discovery concepts into executable code for in silico experimentation. In wet-lab contexts, CRISPR GPT [97] automates guide RNA design, system selection, and protocol drafting, providing an end-to-end pipeline for gene-editing experiments. Biomni [185] similarly supports autonomous hypothesis generation and experimental design by interpreting complex multimodal biomedical data to propose testable biological mechanisms and generate verifiable protocols.

##### Knowledge Graphs and Adaptive Graphs.

Agents also advance discovery by interpreting genomic and structured data with explicit knowledge grounding. GeneAgent [43] enhances gene-set analysis by cross-checking results with biomedical databases, improving reliability in preclinical scenarios. Knowledge graphs provide a basis for auditable reasoning. Biomni [185] performs KG-aware inference across gene-disease-drug-pathway relations to support transparent research decisions. Related approaches combine retrieval-augmented generation with multi-hop KG reasoning [98] and employ generate-verify-revise loops to improve accuracy in interpreting scientific evidence [83].

##### Multimodal Modeling and Imaging Foundations.

In multimodal research, agents integrate imaging with other biomedical data to uncover novel associations. PRISM2 [152], trained on 700,000 whole-slide images and paired reports, provides a foundation for pathology research by enabling zero-shot classification and biomarker prediction, forming a basis for downstream agent pipelines. Building on such foundations, MESHAgents [39] apply multi-agent reasoning to cardiovascular imaging, linking image features to risk factors to improve classification and support causal analysis and explainability. Biomni [185] interprets complex, multi-modal biomedical datasets and autonomously generates experimentally testable protocols.

#### 6.3.2. Medical education and training

AI agents are shifting medical education from static content delivery to interactive, practice-based learning. Large language models support personalized curricula and adaptive learning plans [186], and agentic systems extend this by simulating clinical interactions. By acting as virtual patients, expert coaches, or interdisciplinary colleagues, they create dynamic environments for training communication, reasoning, and team coordination.

##### Clinical Encounters Simulation.

A central application is simulating clinical encounters for skill development. These range from conversational practice (e.g., one agent as a patient and another providing structured feedback) to full diagnostic encounters requiring history-taking and reasoning under uncertainty. Representative examples include ChatCoach [187], which refines consultation skills, systems that support training in sensitive tasks such as breaking bad news [188], and adolescent health education delivered through interactive narrative games [189]. Fidelity is often achieved by grounding simulated patients in illness scripts or de-identified EHR data, with feedback aligned to rubrics such as OSCE checklists [80,190]. Advanced platforms such as AgentClinic [101] extend this approach by allowing learners to interact with multimodal patients, order tests, and make diagnostic decisions, giving instructors a means to identify reasoning gaps.

##### Collaborative and Procedural Training.

Another application focuses on team-based and procedural training. MEDCO [62] creates interdisciplinary environments where agents represent patients, physicians, and radiologists, enabling learners to practice collaboration across roles. SurgBox [82] addresses surgical education by coordinating agents across perioperative phases and acting as a real-time copilot, giving surgeons a controlled environment to rehearse complex procedures.

As these systems become more common, medical educators face new challenges. The focus is shifting from content delivery to high-fidelity simulation design. AI-assisted workflows can help define objectives, generate patient cases, and prepare debriefing plans, reducing effort while maintaining alignment with established training standards [191].

### 6.4. Optimizing healthcare administration

Beyond direct clinical care, healthcare relies on an administrative layer, including hospital managers, regulators, and logistics staff, to maintain operational efficiency and regulatory compliance. These stakeholders oversee workflows such as scheduling, billing, and compliance reporting. For this domain, agentic AI, especially multi-agent systems, can coordinate and execute workflows, automating end-to-end processes such as appointment matching and regulatory compliance tasks. The main risks are operational and legal rather than clinical. This section examines applications at two levels: internal hospital automation, and system-level governance and logistics.

#### 6.4.1. Hospital operational automation

Agentic systems are being adopted to improve hospital operations by automating logistical workflows, digital administration, and financial reporting. These applications aim to ease the burden of routine tasks while supporting more effective use of institutional resources.

##### Hospital Workflow & Operational Efficiency.

Agents can also be used to improve internal hospital operations or resource use. ORDiRS-Agent [145] leverages digital twin representations and reasoning segmentation to analyze operating room workflows from video streams. It decomposes high-level queries into sub-tasks to generate actionable insights on bottlenecks, staffing utilization, and resource occupancy. [192] supports privacy-preserving workflow analysis by converting raw video into de-identified digital twins before event detection. Together, these methods help hospital management identify inefficiencies, optimize staffing, and plan room schedules.

##### Administrative Task Automation.

Some works directly target the automation of backend administrative workflows within healthcare institutions. One framework [8] proposes automating general administrative tasks in healthcare via LLM agents, such as document generation, scheduling, or internal coordination. MedScrubCrew [81] is a multi-agent framework for automated patient–provider matching and scheduling that optimizes resource use across provider availability and patient preferences. These systems reduce staff workload, shorten delays, and improve service capacity.

##### Coding & Reporting.

A further domain is the use of agents for coding, reporting, and regulatory compliance tasks. “Code Like Humans” [193] is a multi-agent solution for medical coding, facilitating revenue cycle management and billing processes with minimal human intervention. A multi-agent approach for International Classification of Diseases (ICD) coding [194] pushes this further in large-scale, automatic ICD assignment. In oncology settings, [195] demonstrates the feasibility of LLMs for registry submission and reporting tasks under real-world constraints. These agents act as intermediaries between raw clinical data and institutional reporting systems, reducing manual work and error rates.

#### 6.4.2. Institutional governance and system logistics

A related direction targets institutional and systemic challenges. Agent-based systems help navigate regulations, monitor compliance, and coordinate inter-institutional logistics. Studies also explore their use in supply chain management, clinical research oversight, and safety standard implementation.

##### Regulation, Policy, and Safety Oversight.

These works target regulators, policy makers, or institutional governance. For example, [196] proposed that the future of LLM-based health apps depends on regulators enforcing safety standards, highlighting the need for governance frameworks to ensure safe deployment in healthcare settings. More broadly, [197] calls for regulatory innovation for generative AI and LLMs in health and medicine, advocating adaptive policies, regulatory sandboxes, and international harmonization. These works emphasize that agent deployment is not purely technical and must be grounded in legal, ethical, and institutional frameworks.

##### Supply Chain, Compliance, and Research Oversight.

Some works address larger-scale institutional or cross-institutional administration. A negotiation agent [198] for medical supply chains integrates LLMs and blockchain to coordinate deliveries, maintain resilience, and handle contracts in uncertain settings. [199] builds an agentic system for assessing medical device compliance across different legal jurisdictions, useful for manufacturers interacting with varied regulatory bodies. [200] proposes an automated protocol adherence system, useful in coordinating research compliance and internal governance. TrialGenie [146] empowers automated design of clinical trial protocols using agentic intelligence combined with real-world data, reducing overhead in research administration.

## 7. Evaluation framework

Evaluating healthcare agents requires a layered framework that links technical accuracy to clinical impact. A rigorous protocol should assess (1) *task and agentic performance*, such as planning and collaboration within established workflows; (2) *simulation, clinical integration, and governance*, i.e., how agents operate safely in clinical environments and comply with institutional standards; and (3) *LLM-as-a-judge*, scalable assessment of open-ended output, validated through expert-aligned meta-evaluation. Together, these layers connect model performance with real-world clinical effectiveness. To provide a structured overview, Table 6 maps evaluation paradigms to application domains, reporting clinician involvement, safety metrics, and documented error types across the 223 reviewed studies.

### 7.1. Task and agentic performance metrics

Traditional *task metrics*—accuracy/F1 for classification [201], exact match for VQA [202], Dice/IoU for segmentation [203], and BLEU/ROUGE/BERTScore for report generation [204]—remain the foundation for evaluating individual components. Recent benchmarks extend this to *multi-step clinical workflows*: AgentClinic [101], MedAgentBench [205], MedAgentBoard [206], and CliBench [207] assess task completion, guideline adherence, and dialogue efficiency in simulated or virtual EHR environments (Table 6 maps these paradigms to application domains).

Building on these workflow-level evaluations, recent work has introduced *agent-specific metrics* that capture not only “what” an agent produces but also “how” the result is obtained. Recent studies consistently adopt the following metrics:

- **Planning Execution Quality** evaluates how consistently an agent executes its plan under constraints such as incomplete information or required tool use, and how well the final outcome aligns with the intended reasoning path [6,101,205]. For example, HealthFlow [208] examines self-evolving planning and tracks how success rates improve with step budgets and converge over iterations, providing a framework for assessing planning quality over time.
- **Tool-Use Quality** evaluates an agent’s ability to orchestrate external tools, e.g., correct API selection, valid parameters, successful execution [101,209]. Vision-based systems such as CT-Agent [37] and CPathAgent [36] assess both tool-use success and the grounding of decisions in visual evidence to ensure faithfulness. MedOrch [209] extends this by providing transparent step traces that facilitate auditing its tool usage.
- **Efficiency and Cost** quantify the computational and interaction resources required by an agent. Key indicators include latency, token usage, and the number of interaction turns needed to complete a task. MedAgentsBench [210] standardizes evaluation across performance, cost, and latency for complex clinical questions. Interactive benchmarks such as MEDIQ [211] additionally assess information-seeking efficiency as a subgoal of agent performance.
- **Collaboration** introduces metrics for evaluating cooperation in multi-agent pipelines. MedAgentBoard [206], for example, reports collaboration gain and planning stability when comparing with alternative approaches.

### 7.2. Simulation, clinical integration, and governance evaluation

Evaluation increasingly extends beyond task accuracy to assess clinical integration and institutional alignment along three tiers. *Simulation-based evaluations* test multi-actor safety in controlled settings, measuring constraint violations and sequential decision success [212] as well as clinician-rated usefulness [213]. *Clinically integrated studies* use guideline concordance [214] and expert agreement on real-world EHR data [215] as proxies for decision quality. *Safety and governance-oriented evaluations* incorporate privacy compliance and policy enforcement as measurable dimensions [216], with recent reviews [217] calling for integrated frameworks that connect model-level metrics with workflow efficiency, safety, and usability. Emerging work has begun to systematize these efforts: collaborative failure taxonomies identify dominant error modes in multi-agent pipelines such as information loss and opinion suppression [218], while dual-track benchmarks jointly quantify safety and clinical effectiveness [219]. Table 6 maps these evaluation tiers to application domains, reporting clinician involvement and safety metrics for each.

### 7.3. LLM-as-a-judge

As clinical tasks become more open-ended, evaluating the quality of generated text increasingly relies on LLMs themselves as evaluators. This shift, often called *LLM-as-a-judge*, aims to assess both semantic accuracy and clinical evidence grounding. Early systems used structured, clinically informed metrics such as RadGraph F1 and RadCliQ [220, 221], which align better with radiologist judgments. Recent work introduces LLM-enhanced evaluators that interpret clinical text directly. GREEN [222] uses an LLM to detect and explain clinically significant errors, providing category-level counts that correlate with expert reviews. GEMA-Score [89] combines structured extraction (NER-based F1) with an LLM scoring module for completeness and readability.

#### Meta-evaluation of LLM judges.

The reliability of LLM judges is a growing concern. Surveys [223,224] identify issues such as prompt sensitivity, bias, and inconsistent scoring, and recommend reporting expert agreement (Spearman or Kendall correlation), prompt robustness, and the use of supporting evidence. To validate, resources such as RadEvalX [225] and ReXrank [226] provide expert annotations and leaderboards to benchmark how well automated metrics, including LLM-based ones, align with human evaluation. Later studies [227] suggest grounding judgments in predefined rubrics, using multiple judges for consensus, and including human audits to reduce inconsistency. In practice, we recommend future papers employing LLM judges should report (1) agreement with clinicians, (2) judging prompts and rubrics, (3) evidence-conditioning protocols, and (4) judging cost relative to human review. These practices improve transparency and reproducibility across agent evaluation.

Ultimately, integrating human-grounded meta-evaluation with automated LLM judging creates a hybrid paradigm that balances scalability and trustworthiness, ensuring that progress in AI agents reflect clinical improvement rather than metric inflation [228]. While promising, large-scale evaluation pipelines still face computational and accessibility constraints. Future work on lighter and open models could make clinical evaluation more practical.

## 8. Discussion: Toward deployment readiness

The rapid growth of healthcare agent research has primarily focused on demonstrating technical capability. However, movement from experimental systems to real-world clinical integration requires a broader lens. Performance improvements on benchmark tasks are not sufficient to establish readiness for deployment in safety-critical environments. Healthcare agents operate within organizational, regulatory, and professional structures that impose constraints extending beyond algorithmic accuracy. To assess the field’s trajectory toward practical integration, we frame the discussion around three interrelated dimensions of deployment readiness. First, *technical justification*: under what conditions does agentic orchestration provide cost-adjusted utility compared with strong non-agent baselines? Second, *translational maturity*: how robust and clinically grounded is the current evidence base supporting these systems? Third, *governance readiness*: how must liability, accountability, and human responsibility boundaries be structured when agents participate in clinical workflows? Together, these dimensions provide a deployment-oriented perspective that complements the preceding technical survey and situates healthcare agent research within the practical constraints of real-world integration.

### 8.1. Technical justification: Cost-adjusted utility

Agentic systems often improve task completion on complex, multi-step problems compared with single-pass generation. However, orchestration introduces additional operational burdens that affect deployability. These include higher and more variable latency, increased token consumption due to iterative invocation, greater infrastructure overhead, and expanded failure surfaces from tool interactions and intermediate reasoning steps. Each added stage increases system dependencies and the potential for error propagation.

In practice, agentic design is justified only when its incremental utility over strong non-agent baselines outweighs these operational costs. Evaluation therefore shifts from isolated accuracy gains to cost-adjusted performance under realistic workload constraints. In healthcare, this assessment is inherently context-dependent, shaped by latency tolerance, resource availability, risk sensitivity, and workflow integration. Industry guidance recommends beginning with the simplest viable design and introducing agentic coordination only when task requirements exceed single-pass capacity [229]. Structured workflows often suffice for predictable tasks, whereas agentic architectures may be warranted when adaptive, tool-mediated reasoning is required.

Empirical evidence quantifying these tradeoffs remains limited but is growing. Recent evaluations show that optimizing solely for accuracy can substantially increase cost without proportional gains in robustness [230]. Observational studies of deployed systems report constrained autonomy such as capped step counts and human checkpoints to manage latency and reduce failure propagation [231]. Healthcarespecific findings further illustrate context dependence: orchestration can improve stability under large-scale batching conditions [232], yet multi-step retrieval and reasoning may introduce substantial latency with only marginal accuracy gains in time-sensitive settings [233].

Overall, systematic cost-aware comparisons between agentic and non-agent baselines remain sparse. Current evidence suggests that agentic coordination can provide meaningful advantages under specific workload and risk conditions, but its benefits are contingent on operational context. Establishing standardized, cost-sensitive evaluation protocols is therefore a critical direction for healthcare agent research.

### 8.2. Translational maturity: Levels of clinical evidence

The reported performance of healthcare agent systems should be interpreted in light of their evaluation context. Different study settings reflect different levels of evidence and translational maturity. To provide a structured assessment of the current literature, we classified studies according to their evaluation setting. *Benchmark* refers to evaluation on standardized datasets or exam-style tasks with predefined inputs and ground truth labels, such as public leaderboards. *Simulation* desscribes evaluation in artificially constructed or highly abstracted environments intended to test capability rather than reflect real clinical data or workflows. *Retrospective clinical data* denotes offline evaluation on previously collected real-world data that does not influence ongoing care. *Pilot deployment* indicates limited integration into clinical workflows under close human supervision. *Prospective study* refers to evaluation conducted under a predefined protocol in live clinical settings involving real patients, where system outputs may affect care.

Using these definitions, we categorized the evidence base by evaluation setting, with the result shown in Table 7. Most studies were conducted in benchmark or simulation environments. Specifically, 103 relied primarily on benchmark datasets and 38 were evaluated in simulated settings. In comparison, 30 used retrospective clinical data, while only 6 pilot deployments and 6 prospective studies were identified. This distribution suggests that the majority of reported performance gains remain pre-deployment. These settings correspond to different levels of evidentiary strength. Benchmark evaluations provide controlled and comparable measurements of task performance. Simulation enables early-stage feasibility testing with limited operational burden. Retrospective studies assess behavior on authentic clinical data but do not evaluate workflow integration or live use. Pilot and prospective studies offer stronger evidence of feasibility within real organizational contexts and clinical responsibility structures.

Taken together, the literature indicates that healthcare agent research remains at an early translational stage. Benchmark and simulation results demonstrate technical potential, yet broader deployment-oriented evaluation is required to establish operational stability, integration feasibility, and real-world clinical value.

### 8.3. Governance readiness: Liability and human accountability

Healthcare agents operate in environments where decisions directly affect patient safety, legal responsibility, and institutional compliance. In such settings, liability and accountability are foundational design constraints. The level of autonomy granted to an agent shapes how responsibility must be distributed and formalized within clinical workflows.

When healthcare agents incorporate tool use and system-level integration, they may move beyond generating recommendations to performing actions such as writing to medical records, initiating clinical tasks, or invoking external services. In such settings, liability is shaped by how authorization and execution are governed within existing professional and regulatory frameworks. Responsibility must be explicitly tied to defined workflow roles and decision points.

Accountability is typically maintained through separation between recommendation, authorization, and execution. Agents may produce diagnostic suggestions, draft documentation, generate analytic outputs, or prepare workflow actions. However, a licensed clinician or designated operational owner remains the decision-maker of record. Clinical responsibility lies with the professional who approves diagnostic and treatment decisions. Documentation accountability rests with the individual whose authorization finalizes the record. In administrative and research contexts, responsibility remains with the operational lead or principal investigator who configures, supervises, and approves system actions.

Under prevailing regulatory and professional norms, identifiable human accountability is required in clinical decision-making and system deployment [275–278]. Healthcare agents therefore operate within delegated scopes defined by authorization gates, auditability requirements, and institutional oversight. Across the reviewed systems, high-risk or irreversible actions consistently retain human approval, reflecting the safety-critical and regulated nature of healthcare practice.

## 9. Future challenges and opportunities

The preceding discussion evaluated the current deployment readiness of healthcare agents across technical justification, evidentiary maturity, and governance structures. Despite rapid architectural progress, significant barriers remain before widespread clinical integration can be achieved. These barriers arise from the end-to-end nature of agentic systems, which span perception, reasoning, tool invocation, and workflow execution within safety-critical environments.

**Fig. 5.**
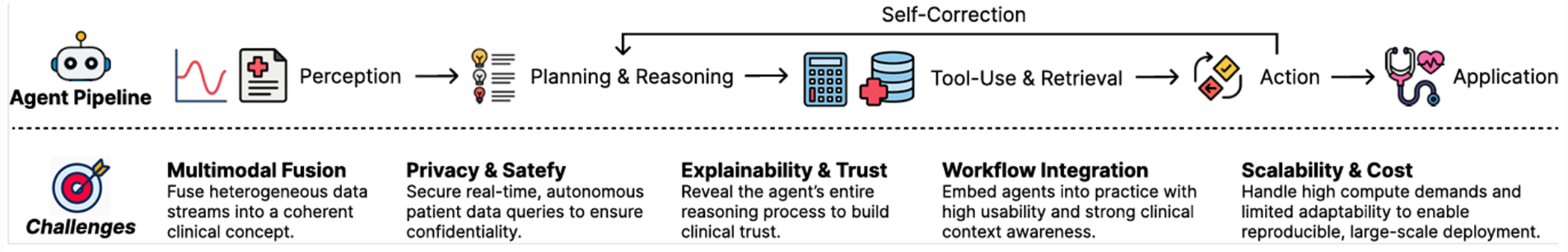
A high-level overview of key challenges facing AI agents in healthcare.

Fig. 5 illustrates a typical healthcare agent operating across the clinical pipeline: processing multimodal inputs, performing structured planning and reasoning, accessing external tools and knowledge sources, and interacting with institutional systems. At each stage, distinct vulnerabilities emerge, including multimodal alignment errors, propagation of intermediate reasoning failures, real-time data governance risks, insufficient decision transparency, workflow integration friction, and scalability constraints under operational workloads.

### 9.1. Reliable fusion across modalities

Effective multimodal fusion is essential to clinical reasoning and decision-making. In sequential decision systems, fusion errors are not simple misclassifications—they can propagate into a chain of faulty downstream actions, from ordering contraindicated tests to missing critical interventions. The challenge lies not only in integrating heterogeneous data streams but also in bridging the semantic gap between raw signals and clinically meaningful concepts [49,86]. Moreover, these systems must remain reliable under incomplete information, which requires more than data imputation [234]. They must reason under uncertainty and proactively plan missing data acquisition, as a clinician would during diagnosis.

### 9.2. Clinical workflow integration

Integrating advanced AI into clinical practice requires moving from decision support to active collaboration. Systems that act and interact must maintain strong contextual awareness to ensure their behavior is safe and relevant, such as recognizing the urgency of an ICU compared with the pace of an outpatient clinic [160]. Achieving this balance requires careful human-AI interaction design rooted in *mixed-initiative interaction* [279]. The system should dynamically negotiate control with users, taking initiative when appropriate and deferring when human judgment is essential [280,281].

The collaborative potential of agentic systems depends on interoperability. Their ability to perceive context and execute tasks relies on stable communication with diverse hospital IT systems; when integration is fragile, deployment fails. Adoption also depends on usability: clinicians should be able to operate these systems without programming skills or complex setup [282,283]. Interfaces should support low-friction workflows, role-appropriate abstractions, and safe defaults that reduce cognitive and operational load.

### 9.3. Governance, safety, and regulatory compliance

Healthcare agents operate in safety-critical settings where errors can directly affect patient outcomes. As these systems move from advisory tools to action-enabled components within clinical workflows, governance must address multiple dimensions, including privacy, accountability, risk management, and regulatory compliance. When agents access records, generate documentation, or invoke external systems, responsibility must remain clearly assigned. Clinical accountability resides with licensed professionals who approve diagnostic and treatment decisions. Documentation responsibility rests with the signer of record. In operational and research contexts, designated owners such as clinical leads or principal investigators retain oversight of system configuration and use. This structure aligns with prevailing regulatory frameworks that require identifiable human responsibility for clinical decisions and system deployment [275–278].

Beyond privacy, regulatory feasibility depends on whether agentic systems meet established medical device requirements. In the European context, software that informs diagnosis or treatment may be regulated under the EU Medical Device Regulation (EU) 2017/745, particularly Rule 11 governing software classification. Systems whose outputs influence clinical decisions may therefore qualify as Software as a Medical Device (SaMD), triggering obligations for risk management, technical documentation, traceability, lifecycle control, and post-market surveillance [284,285].

Safe deployment accordingly requires enforceable architectural safeguards, including gated execution for higher-risk actions, auditable logs, provenance tracking, and least-privilege access controls [286]. Runtime governance mechanisms must complement privacy-preserving training methods [244]. While compliance is conceptually achievable, the adaptive, tool-using, and multi-step nature of agentic architectures introduces challenges for explainability, change management, and continuous learning under current regulatory regimes. These regulatory and safety requirements remain a central barrier to routine clinical adoption.

### 9.4. Security threat models and mitigations

Tool-enabled healthcare agents introduce security risks that extend beyond conventional data protection concerns. Because these systems retrieve clinical information, call external tools, and perform workflow actions, security failures can affect clinical decisions. Key threat models include prompt injection, retrieval-enabled data exfiltration, malicious tool outputs, and adversarial clinical content [287–290]. These attacks may override system policies, expose sensitive information, or propagate incorrect results into downstream reasoning.

Mitigations focus on constraining execution and increasing traceability. Sandboxing, least-privilege capability scoping, and allowlists restrict tool access [286,291,292]. Provenance verification and policy engines support authorization and auditability [293,294]. Continuous monitoring and red-teaming improve detection of unsafe behaviors [295,296]. Each safeguard introduces trade-offs in flexibility and performance, requiring careful calibration to clinical context [297].

### 9.5. Explainability for trustworthy AI

To earn clinical trust, explanations must move beyond predictions to reveal the reasoning process behind each recommendation. When systems initiate actions, clinicians need transparency into their goals, alternative strategies, and the rationale for their final decisions [79,85]. Such transparency is essential for safely delegating clinical tasks. Trust also depends on a system’s ability to communicate uncertainty. A reliable collaborator should recognize its knowledge limits and express confidence not only in its findings but also in the expected outcomes of its recommendations. This “explainable uncertainty” helps calibrate clinicians’ reliance on the system and supports safe, effective human-AI collaboration [298].

### 9.6. Adaptability and reproducibility for scalability

The substantial computational cost of developing advanced systems poses a scalability challenge closely tied to generalization. High upfront investments are difficult to justify if systems remain brittle and fail to adapt to new clinical environments, a common issue known as domain shift. A promising approach to improving scalability is to move beyond this static design, requiring architectures that are both powerful and adaptable. For example, self-evolving systems can discover new tools, integrate emerging knowledge, and refine their strategies over time [72].

Scalability also depends on reproducibility. When code, models, or datasets are unavailable, proprietary artifacts and opaque configurations prevent replication and hinder cross-site benchmarking. Together, adaptability and reproducibility determine whether these technologies can scale efficiently and equitably across diverse healthcare settings [56,257].

## 10. Conclusion

This survey has reviewed the current landscape of AI agents in healthcare through an analysis of more than 200 recent studies. We proposed a taxonomy spanning perception of clinical modalities, agent capabilities and architectures, application domains, and evaluation approaches. Beyond mapping technical advances, we examined the deployment readiness of healthcare agents across cost-adjusted utility, evidentiary maturity, and governance accountability.

Our analysis indicates that clinical requirements for safety, reliability, and accountability are shaping agent design in ways that diverge from general-purpose systems. While benchmark and simulation results demonstrate strong technical potential, translational maturity remains limited, with relatively few systems evaluated in real-world clinical settings. Technically, robust multimodal integration, seamless workflow compatibility with clinical IT, and scalable orchestration remain open challenges. At the governance level, safeguarding data privacy and achieving process-level explainability are necessary to build trust in clinical settings.

Advancing healthcare agents toward responsible integration will require coordinated progress across technical validation, deployment-oriented evaluation, and institutional governance. The trajectory of research suggests a shift toward structured human–AI collaboration, emphasizing systems that are not only autonomous, but verifiable, accountable, and safely embedded within patient care workflows. This survey provides a structured foundation for guiding that transition.

## Figures and Tables

**Table 1:** Statement of significance.

Problem or Issue	The high-stakes, safety-critical nature of healthcare makes general-purpose agentic frameworks inadequate, creating significant challenges in trust, reliability, and clinical alignment.
What is Already Known	Research on healthcare agents is growing fastly, yet existing reviews are often narrow, focusing only on specific modalities or lacking systematic analysis of domain-specific constraints.
What This Paper Adds	This survey synthesizes over 200 recent studies into a holistic taxonomy. It links multi-modal perception and core agent capabilities to a stakeholder-centric application ecosystem, while critically addressing evaluation and governance.
Who would benefit from the new knowledge in this paper	Clinicians, AI researchers, and healthcare administrators seeking to understand, develop, or regulate autonomous medical systems.

**Table 2:** Perception modalities, input data, perception methods, agent roles, and representative systems in healthcare agents.

Modality	Input data	Perception methods	Representative roles	Representative systems
Natural language conversation	Dialogues, QA, therapy text	Text understanding	Clinician	DoctorAgent-RL [27] MedAgents [28] CACTUS [29]
Electronic health records	Structured EHR tables, schemas	Data analysis → Text understanding	Data analyst	EHRFlow [30] TrustEHRAgent [31]
Clinical notes	Longitudinal text, FHIR mapping	Temporal extraction → text understanding	Diagnostic assistant	Infherno [32] CARE-AD [33]
2D medical imaging	CXR, WSIs	Image encoding → token patching	Radiologist, pathologist	MedRAX [34] PathChat+ [35] CPathAgent [36]
3D medical imaging	CT/MRI volumes	Volume encoding → token patching	Radiologist	CT-Agent [37] AgentMRI [38] MESHAgents [39]
Time-series signals	ECG, PPG, wearables	Statistical analysis → text understanding	Computational analyst	PHA [40] LLM-PPG [41]
Genomics and biomarkers	Gene sets, mutations, omics	KG reasoning → statistical analysis → text understanding	Bioinformatics analyst	HEAL-KGGen [42] GeneAgent [43] AI-HOPE [44]
Audio	Speech, emotion cues	Speech-to-text → text understanding, waveform analysis	Empathetic listener	ANNA [45] Survey Agent [46]
Multi-modality	Text, image, signal, omics	Combination of above	Coordinator, tool orchestrator	MAM [47] MedAgent-Pro [48] MMedAgent [5]

**Table 3:** Overview of agent architectures, their primary capabilities, representative systems, and key contributions.

Architecture	Capability	Representative systems	Key contribution
Knowledge Retrieval	Grounding agent decisions in curated evidence	AMG-RAG [51] Path-RAG [52] AI-VaxGuide [53]	Automates integration of existing knowledge into generation to reduce hallucinations and improve diagnostic accuracy over LLM-only baselines.
Memory	Maintaining longitudinal context	AMC [54] REMI [55] EHRAgent [6]	Maintains explicit memory of patient-history or experiment-history to improve decision-making.
Planning and Reasoning	Decomposing tasks, reasoning decisions	STELLA [56] CT-Agents [37] MeNTi [57]	Implements explicit task-decomposition and structured reasoning, integrating tool use and intermediate feedback to iteratively refine plans and conclusions.
Tool Use	Extending the agent’s capabilities by integrating and selecting external software tools	GeneAgent [43] DrugAgent [58] AgentMD [59]	Incorporates and dynamically selects domain-specific tools into the agent architecture.
Simulation	Creating and maintaining a controlled clinical persona for the agent	Talk2Care [60] BehaviorSFT [61] Medco [62]	Develops and evaluates methods to encode and enforce agents’ role identity.
Multi-Agent Systems	Enabling collaboration between multiple agents	MAC [63] MAM [47] PathChat+ [35]	Routes tasks to domain-specialized agents while using coordination algorithms to formalize team behaviors.

**Table 4:** Practical autonomy scale for healthcare agents and recommended minimum oversight controls by risk tier. Risk tiers reflect escalating potential for patient harm: R0 (administrative and operational), R1 (patient-facing informational), R2 (clinical decision support), R3 (direct intervention or time-critical action).

Autonomy	Operational definition	Risk tier(s)	Suggested minimum oversight and controls
A0	**Suggest-only.** The agent generates recommendations, summaries, or explanations, but does not execute actions in external systems.	R0–R2	**Accountability**: A human retains final responsibility for decisions. **Transparency**: The system communicates key uncertainty and limitations. **Provenance**: Claims are traceable to supporting evidence. **Safety and audit**: Outputs are filtered for harmful content and logged for review.
A1	**Draft-for-sign-off.** The agent produces drafts that can be used only after explicit human approval; no system write operations without sign-off.	R0–R2	**Sign-off**: Drafts require explicit human approval before use. **Traceability**: Versions and edits are retained for audit. **Separation**: Draft and approved content are clearly distinguished. **Structured review**: High-impact drafts follow a standardized checklist.
A2	**Execute-with-gates.** The agent can call tools and perform bounded actions, but only through gates, such as policy checks and explicit authorization for sensitive steps.	R1–R3	**Least privilege**: Tool access is narrowly scoped. **Validation**: Actions pass rule-based checks before execution. **Authorization**: High-risk actions require explicit human authorization. **Monitoring**: Execution is monitored with alerts, escalation, and rollback when feasible.
A3	**Autonomous execution.** The agent can autonomously complete multi-step tasks without step-by-step human approval.	R2–R3	**Bounded scope**: Autonomy is restricted to predefined tasks and contexts. **Continuous monitoring**: Real-time supervision with an immediate stop mechanism. **Accountability**: Comprehensive action logging supports post-hoc review. **Governance**: Regular safety evaluation and incident reporting are required.

**Table 5:** Overview of application domains, typical autonomy levels (A0–A3; see Table 4), agent roles, key capabilities, and representative systems for healthcare agents.

Application domain (Stakeholder)	Autonomy	Agent role	Key agentic capability	Representative systems
Diagnosis & Decision Support (Doctors)	A0–A1	Diagnostic Assistant MDT Simulator	Planning & Reasoning Tool-Use Multi-Agent Systems	AgentMD [59] TxAgent [140] CT-Agent [37] ColaCare [126]
Clinical Documentation (Doctors)	A1	Clinical Scribe Reporter	Knowledge Retrieval (RAG) Tool-Use (FHIR/API)	Infherno [32] PathChat+ [35] FRAME [141]
Workflow Automation (Doctors)	A1–A2	Digital Assistant Executor	Planning (NL-to-Code) Tool-Use	EHRFlow [30] CDR-Agent [142] SurgBox [82]
Mental Health & Counseling (Patients)	A0–A1	Therapeutic Cunselor	Simulation (Persona) Memory (Long-Term)	AutoCBT [143] CAMI [109] MAGI [144] VChatter [112]
Patient Education (Patients)	A0	Health Coach Educator	Knowledge Retrieval (RAG) Memory Simulation (Persona)	AI-VaxGuide [53] CareCall [76]
Biomedical Research (Researchers)	A1–A2	Research Assistant	Planning Tool-Use Knowledge Retrieval	Stella [56] BioScientistAgent [103] DrugAgent [58]
Medical Education (Educators)	A0–A1	Virtual Patient Colleague	Simulation (Persona) Multi-Agent Systems	AgentClinic [101] MEDCO [62]
Hospital Automation (Administrators)	A2	Administrative Coordinator	Multi-Agent Systems Planning	MedScrubCrew [81] ORDIRS-Agent [145]
Institutional Governance (Administrators)	A1	Compliance Logistics Analyst	Knowledge Retrieval (RAG) Planning	TrialGenie [146]

**Table 6:** Evaluation landscape across application domains. For each domain we report the dominant evaluation paradigms, clinician involvement, representative safety or governance metrics, and documented error or failure types, aggregated from 223 reviewed studies. Risk tiers follow the definitions in Table 4.

Application domain	Risk	Eval. paradigm(s)	Clinician involvement	Safety/Governance metrics	Error/Failure types
Diagnosis & Decision Support	R2	Benchmark, Simulation, Retrospective	Expert agreement, guideline concordance	Guideline adherence, diagnostic accuracy vs. expert panel	Misdiagnosis, hallucinated findings, missed critical conditions
Clinical Documentation	R1–R2	Benchmark, Retrospective	Clinician sign-off, correction rate	Factual consistency (RadGraph F1, GREEN)	Hallucinated content, omission of key findings
Workflow Automation	R0–R1	Benchmark, Pilot	Physician-authored task design	EHR query correctness, privacy-preserving access	Invalid API calls, data retrieval failure
Mental Health & Counseling	R1–R2	Benchmark, Simulation	Therapist rating, psychiatric protocol fidelity	Crisis escalation triggers, therapeutic alliance (ESC-Judge)	Inappropriate response, missed crisis signals, demographic bias
Patient Education	R1	Benchmark, Pilot	Content accuracy review	Medical accuracy, guideline alignment	Misinformation, culturally inappropriate guidance
Biomedical Research	R0	Benchmark, Simulation	Limited evidence	Reproducibility, code execution correctness	Hallucinated citations, invalid tool invocation
Medical Education	R0–R1	Simulation	OSCE checklist, instructor review	Pedagogical validity, patient fidelity	Unrealistic patient behavior, misleading feedback
Hospital Automation	R0	Benchmark, Retrospective	Limited evidence	Scheduling accuracy, coding compliance	Incorrect ICD codes, appointment mismatch
Institutional Governance	R0–R1	Retrospective, Pilot	Compliance review	Regulatory adherence, policy enforcement	Protocol deviation, regulatory misclassification

**Table 7:** Reality check reference table.

Category	Papers
benchmark	[12,14,18,28,31,33,34,38,40,42,47,48,52,53,57,61,66,67,69,71,77,81,83–85,87,90,95,99–102,108,116,121–123,125,126,128,130,131,133,134,138,140,146,149,151,153,156,157,160–162,173,177,182,191–194,199,201,202,204–209,211,214,222,225,226,233–257]
simulation	[8,15,27,29,32,50,64,68,80,88,101,109,111–113,116,120,135,148,155,158,164,165,167,172,178,181,185,188,198,235,258–264]
retrospective clinical data	[7,33,49,63,86,106,127,137,141,142,145,147,150,152,153,159,169,171,189,195,200,215,217,265–271]
pilot deployment	[45,110,148,180,213,272]
prospective study	[41,92,99,118,273,274]

## Data Availability

No data was used for the research described in the article.
